# Diminishing storage returns of reservoir construction

**DOI:** 10.1038/s41467-023-38843-5

**Published:** 2023-06-13

**Authors:** Yao Li, Gang Zhao, George H. Allen, Huilin Gao

**Affiliations:** 1 Zachry Department of Civil and Environmental Engineering, Texas A&M University, College Station, TX USA; 2 Department of Global Ecology, Carnegie Institution for Science, Stanford, CA USA; 3 Department of Geosciences, Virginia Polytechnic and State University, Blacksburg, VA USA; 4 Present Address: Chongqing Jinfo Mountain Karst Ecosystem National Observation and Research Station, School of Geographical Sciences, Southwest University, Chongqing, China; 5 Present Address: Key Laboratory of Water Cycle and Related Land Surface Processes, Institute of Geographic Sciences and Natural Resources Research, Chinese Academy of Sciences, Beijing, China

**Keywords:** Hydrology, Environmental impact

## Abstract

Surface water reservoirs are increasingly being relied upon to meet rising demands in the context of growing population and changing climate. However, the amount of water available in reservoirs (and the corresponding trends) have not been well quantified at the global scale. Here we use satellite observations to estimate the storage variations of 7245 global reservoirs from 1999 to 2018. Total global reservoir storage has increased at a rate of 27.82 ± 0.08 km^3^/yr, which is mainly attributed to the construction of new dams. However, the normalized reservoir storage (NS)—the ratio of the actual storage to the storage capacity—has declined by 0.82 ± 0.01%. The decline of NS values is especially pronounced in the global south, while the global north mainly exhibits an NS increase. With predicted decreasing runoff and increasing water demand, these observed diminishing storage returns of reservoir construction will likely persist into the future.

## Abstract (web summary)

Despite the continually increasing global reservoir storage, the normalized storage—the ratio of the actual storage to the storage capacity—has declined over the past two decades, indicating diminishing storage returns from reservoir construction.

## Introduction

Although global reservoirs have a much smaller total capacity compared to natural lakes, their flow regulation represents the most intensive human-induced alteration of the hydrological cycle^1–5^. The 20th Century witnessed a massive dam construction boom, first starting in North America and then spreading to the rest of the inhabited world. Behind these dams, sprawling reservoirs have fundamentally enhanced our ability to manage Earth’s freshwater resources^6–8^, but have also imposed adverse environmental and social effects^9–13^. After a decline in growth during the 1990s^14^, hundreds of large new dams have been added in Asia, Africa, and South America^3^. In addition, over 3700 hydropower dams (each over 1 MW in capacity) are being planned or are under construction as of 2014, most of which are in developing countries^15^. With water scarcity intensified by both climate change^16–19^ and increasing water demand^20^, reservoir water availability is essential for sustainable development^21^. Yet dam construction and reservoir operations are rarely coordinated amongst countries despite nearly half of all land being covered by international river basins^22^. To best inform future decision-making related to global surface water management, the storage conditions of reservoir impoundments—particularly those newly constructed—should be carefully evaluated.

However, knowledge about the long-term variation of reservoir storage is very limited at a global scale. In situ measurements of reservoir storage are often not shared, especially across international river basins^23^. Land-surface and hydrologic models produce highly uncertain storage estimates, largely due to the lack of reservoir operations/management information^24–26^. By relating reservoir surface area and elevation values, satellite remote sensing provides a viable alternative for monitoring storage^27–31^. Although recent studies have quantified long-term surface area time series values and seasonal elevation variations of reservoirs globally^5,32^, reliable storage estimations have only focused on reservoirs built before 1999 (hereafter referred to as “pre-1999 reservoirs”)^27–31^.

**Fig. 1:**
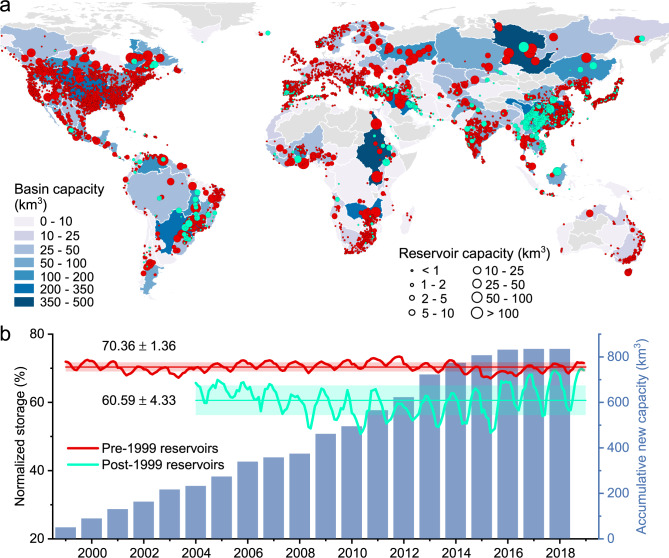
Comparison of normalized storage (NS) variations of global pre- and post-1999 reservoirs. **a** Locations of the global reservoirs (with pre-1999 reservoirs in red and post-1999 reservoirs in cyan). **b** Comparison of the NS values of global pre- and post-1999 reservoirs (excluding regulated natural lakes), along with the accumulative storage capacity of the post-1999 reservoirs.

Here, we develop the Global Reservoir Storage (GRS) dataset to evaluate the conditions of global reservoir impoundments—particularly reservoirs constructed after 1999 (hereafter referred to as “post-1999 reservoirs”). We built GRS using multi-source satellite data by converting monthly reservoir water areas from the Global Reservoir Surface Area Dataset (GRSAD)^32^ to monthly storage values using bathymetric maps^33^ and, where necessary, an improved bathymetry simulation method^34^ (see “Methods”). The GRS dataset is the most comprehensively validated long-term storage record for all of the consistently mapped reservoirs in the Global Reservoir and Dam Database (GRanD)^3^, and represents a major advancement in tracking global reservoir storage conditions. GRS is comprised of storage records from 1999 to 2018 for 7245 reservoirs (6658 km^3^ total capacity) as reported in GRanD^3^ (Fig. 1a). While post-1999 reservoirs only account for 7.14% of the number of reservoirs, their combined storage contributes to 14.34% of total global capacity (834.78 km^3^).

## Results and discussion

We introduce “normalized storage” (NS), a new term defined as the ratio of the actual storage from a group of reservoirs to the storage capacity of these same reservoirs. The NS offers a unique flexibility for quantifying and comparing the storage returns of impoundment from different groups of reservoirs across global, continental, and basin scales. This makes regions with different storage capacities comparable, and it is not affected by the increased storage from new reservoirs. The use of NS allows us to: (1) split the pre- and post-1999 reservoirs and directly evaluate the behaviors of newly constructed reservoirs; (2) compare the NS trends with the actual storage trends for new insights; and (3) assess reservoirs grouped by different functionalities (e.g., hydropower, irrigation).

**Fig. 2:**
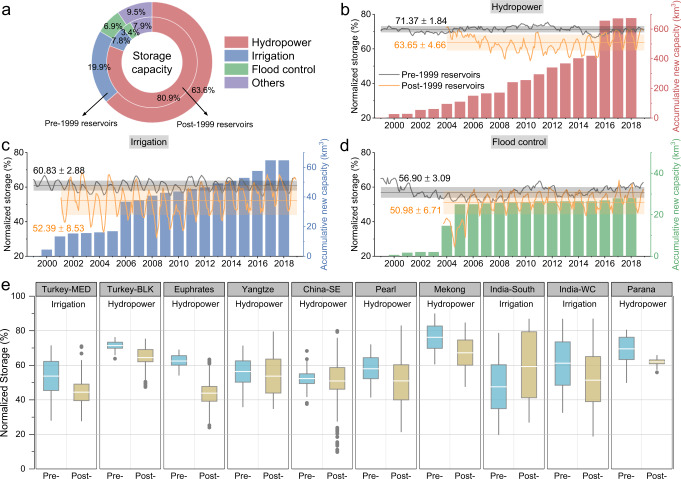
Comparison of normalized storage (NS) in terms of reservoir function at global and basin scales. **a** Reservoir storage capacity proportion corresponding to primary function for pre-1999 (outer ring) and post-1999 (inner ring) reservoirs globally. **b**–**d** Comparison of the NS values of global pre- and post-1999 reservoirs (excluding regulated natural lakes) with primary functions of hydropower, irrigation, and flood control, along with the accumulative storage capacity of the post-1999 reservoirs. **e** Comparison of the NS values of pre- and post-1999 reservoirs (excluding regulated natural lakes) for ten basins that have more than five post-1999 reservoirs with the same primary function. Box ranges represent the upper and lower quartiles, whiskers extend to 1.5 times the interquartile range, and outliers are denoted as dots. The statistical results are summarized in Supplementary Table 1.

While the newly constructed reservoirs have contributed to a steady increase in global storage capacity, their storage returns (in terms of NS) are found to be smaller than those built in the 20th century (over the period of 1999–2018). The NS values of the post-1999 reservoirs are significantly lower (and with larger seasonal variations) than the pre-1999 ones, at 60.59 ± 4.33% and 70.36 ± 1.36%, respectively (Fig. 1b). The NS values also depend on reservoir function. For instance, the NS values in reservoirs whose primarily function is hydropower are generally higher than those whose primarily function is irrigation or flood control. Therefore, we further compared the NS values for pre-1999 and post-1999 reservoirs in terms of reservoir function, at both global and basin scales. Regardless of the function and/or spatial scale, all results lead to the same conclusion—that post-1999 reservoirs have lower NS levels, but larger seasonal variations, than pre-1999 reservoirs (Fig. 2). It is worth noting that there are many social-economic benefits to building reservoirs (e.g., hydropower generation, flood reduction, water supply, and recreation), but here we are framing the returns only in terms of normalized water storage.

**Fig. 3:**
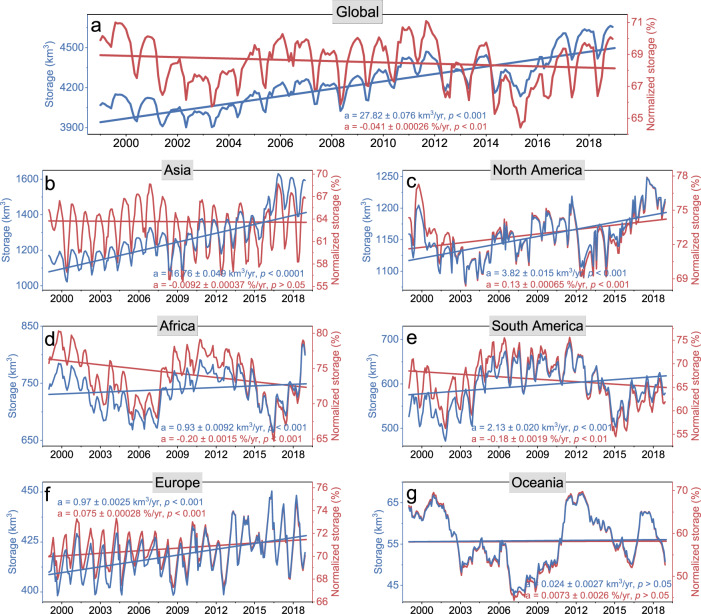
Monthly reservoir storage and normalized storage (NS) variations at the global and continental scales from 1999 to 2018. **a** Global, **b** Asia, **c** North America, **d** Africa, **e** South America, **f** Europe, and **g** Oceania.

The global total reservoir storage shows a nearly continuous increase during the last two decades (Fig. 3), with a mean value of 4236.32 ± 181.64 km^3^ (mean ± std) and a growth rate of 27.82 ± 0.08 km^3^/yr (Supplementary Table 2). This increase is primarily attributable to the construction of new reservoirs at a mean rate of 41.61 km^3^/yr (Supplementary Fig. 1). Unlike the total storage, the global NS has a significant decreasing trend (at a rate of −0.82 ± 0.01%/20 yr). The contrasting storage and NS trends indicate that the recent storage returns of global reservoir construction have been declining since the start of the 21st century.

At the continental scale, this decreasing normalized storage is particularly evident in Asia, Africa, and South America, which are the continents where most of the newly constructed reservoirs are located (Fig. 1a). Asia holds the largest number of reservoirs (2352) and the highest storage capacity (2386.78 km^3^) of any continent. It also has the most rapid storage increase with a growth rate of 16.76 ± 0.05 km^3^/yr, accounting for 60.04% of the global trend from 1999 to 2018. This increase is mainly attributed to new reservoir construction. While the NS of all Asian reservoirs combined has a decreasing trend (−0.18 ± 0.01%/20 yr), the NS value of the pre-1999 reservoirs shows a significant increase (1.30 ± 0.01%/20 yr), which suggests that the overall reduction of NS is attributed to the post-1999 reservoirs. Both Africa and South America show increasing storage but decreasing NS. Their storage growth is primarily driven by newly impounded reservoirs. However, the pre-1999 reservoirs in Africa (−3.81 ± 0.03%/20 yr) and South America (−4.28 ± 0.04%/20 yr) have experienced significant NS drops, which is the driver for the overall NS decrease in these two continents (−3.99 ± 0.03%/20 yr and −3.53 ± 0.04%/20 yr, respectively). For the other three continents—which are dominated by developed countries, and have few post-1999 reservoirs—the storage and NS trends coincide. North America and Europe particularly exhibit significant increasing trends in NS, which together offset the overall global decreasing NS trend. Note that the decreasing NS trends are conservative, as we did not consider the impacts of sedimentation when conducting the trend analysis (see Methods).

**Fig. 4:**
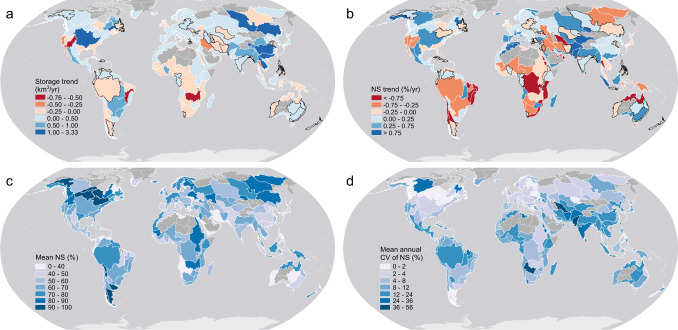
Reservoir storage and normalized storage (NS) variations from 1999 to 2018 at the basin scale. **a** Storage trends, **b** NS trends, **c** Long-term mean NS, and **d** Mean annual coefficient of variation (CV) of NS. Note that with the basins shown in (**a**) and (**b**), the significant trends (*p* < 0.05) are delineated in white, and the non-significant trends (*p* > 0.05) are delineated in black. The number of reservoirs and the total storage for each basin are shown in Supplementary Fig. 2.

Basin-scale reservoir storage information is essential for managing local water resources and assessing changes to the hydrological cycle, yet this knowledge is lacking at the global scale—particularly for transboundary river basins^35^. Thus, we evaluate the storage variations at the basin scale (Fig. 4). The majority of the Earth’s basins experienced storage growth (Fig. 4a). Asian basins have the most storage growth, while basins in southern Africa have suffered from storage losses. The highest increase is found in the Yangtze River Basin (3.34 ± 0.01 km^3^/yr), which is characterized by the most intensive dam construction activities in Asia (e.g., Three Gorges Dam)^15^. The fastest storage decline occurred in the Colorado River Basin (−0.62 ± 0.003 km^3^/yr), due to the combined effects from an extended drought since 2000^36^ and increasing water use^37^. With regard to NS (Fig. 4b), decreasing trends predominate—especially over the Southern Hemisphere, including those areas in South America and southern Africa. The basins with increased NS are mainly in East Asia, Europe, and North America. However, fewer basins in Asia show significant increases in NS compared to storage—and the NS increases in European and North American basins are generally weak.

To assess the overall reservoir water availability and stability, we also examined the long-term mean and variation of the NS values from 1999 to 2018 at the basin scale (Fig. 4c, d). The reservoirs in high-latitude regions (e.g., the Great Lakes and Siberia) have relatively high NS values. This is attributable to the fact that these northern reservoirs are less affected by human activities due to low population density. On the other hand, the basins in South and Southeast Asia (e.g., the Indus and Yangtze basins) have low NS levels likely because of the high water demand driven by the large populations in these areas. Moreover, the annual mean coefficient of variation (CV) of the NS values (Fig. 4d) indicates that the reservoirs in high-latitude areas—as well as those in Europe and North America—are relatively stable, with comparatively small dynamics. In contrast, the Asian reservoirs—with the exception of those in high-latitude regions—show large intra-annual variability. The large NS variability in the Amazon basin is attributable to the extensive damming of highly seasonal Amazonian rivers^38^. Finally, the basin with the highest annual CV value—the Indus basin in India—has experienced substantial groundwater depletion^39^, suggesting that surface water and groundwater levels have both been dominated by human activities in response to the region’s severe water shortage.

**Fig. 5:**
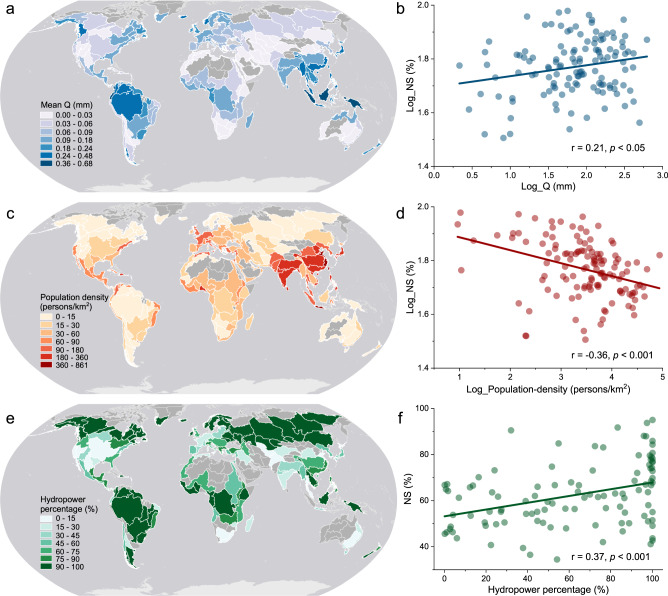
The potential drivers of normalized storage (NS) patterns at the basin scale. **a** Long-term mean runoff (Q) values from 2000 to 2018, **c** Mean population density (2000–2020), and **e** Storage capacity percentage of hydropower reservoirs. Plots **b**, **d**, and **f** show the respective correlations between the metrics to the left (shown in **a**, **c**, and **e**) along with the long-term mean NS values (2000–2018) for global basins that contain more than five reservoirs. Note that a log transformation was applied to the runoff and population data to correct for heteroskedasticity (see “Methods”).

**Fig. 6:**
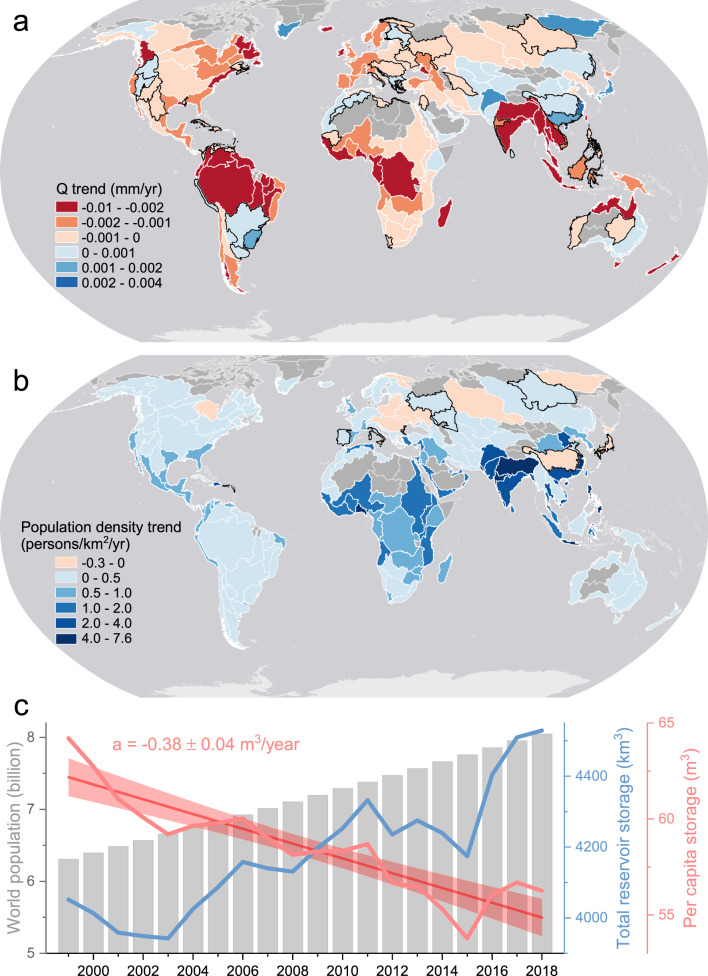
Global water stress under population growth. **a** Runoff (Q) trends from 2000 to 2018 at the basin scale, with the significant trends (*p* < 0.05) delineated in white and the non-significant trends (*p* > 0.05) delineated in black. **b** Population density trends from 2000 to 2020 at the basin scale, with the significant trends (*p* < 0.05) delineated in white and the non-significant trends (*p* > 0.05) delineated in black. **c** The world population, global reservoir storage, and per capita storage from 1999 to 2018. Shading illustrates the 95% confidence intervals for the best-fit linear trends, and ʻaʼ represents the trend value. The world population data were collected from the World Bank (https://data.worldbank.org/indicator/SP.POP.TOTL), and the annual total reservoir storage values were derived by averaging the monthly Global Reservoir Storage dataset. The per capita storage is the ratio of global reservoir storage and world population.

Reservoir NS is impacted by multiple factors, including upstream runoff, population density, and reservoir function—which directly affect reservoir storage values through inflow, demand, and operation (see “Methods” and Fig. 5). The global runoff, especially in tropical regions, suggests a considerable decrease during the last two decades (Fig. 6a). This suggests that declines in runoff may be the driver of the observed trends (of decreasing NS) in reservoirs with large storage variations. The decreasing trends in runoff are most significant in South America followed by Africa, which contribute to the reduction of NS values in these regions (Fig. 6a). These trends may exacerbate already stressed reservoirs where NS is significantly decreasing (Fig. 4b). In the last two decades, global population growth is outpacing increases in reservoir storage, causing a significant decrease in the per capita storage (−0.38 ± 0.04 m^3^/yr, Fig. 6c). If regionally declining runoff persists into the future, it will likely add considerable pressure on global reservoirs for meeting the anticipated rising demands on water resources. In particular, we expect that regions in Africa and South Asia will experience increasing water shortages under the predicted extensive decrease in runoff^16,40,41^ and increase in population (Fig. 6). Special attention should be paid to reservoir water management strategies in these hotspots of water scarcity^39,42^.

Our analysis reveals that the global reservoir normalized storage (i.e., NS) has significantly declined in the 21st century despite an increase in total storage due to the construction of new reservoirs. The changes mainly occurred in South America, and Africa, where the world’s developing countries are located. However, these negative trends have been weakened at the global scale due to the significant increasing NS in the Global North—North America (2.61 ± 0.01%/20 yr) and Europe (1.50 ± 0.01%/20 yr). In particular, the NS values of the post-1999 reservoirs are much smaller than those of the pre-1999 ones. Asia, South America, and Africa are the primary “hotspots” where most future dam construction activities are planned, with Brazil, China, and the Democratic Republic of Congo taking the lead^15^. However, future development of new reservoirs likely will not alleviate the water stress caused by increasing municipal and industrial water demand in south Asia (e.g., India) and southeast Asia (e.g., China). In South America and Africa, the reduced NS values are mainly associated with the large decreases in runoff trends. The results from this study highlight the challenges of resolving finite water resources through reservoir regulation—particularly in developing countries, where the storage returns from these impoundments are diminishing. These findings offer a new perspective for reevaluating the socio-economic benefits of new reservoir construction, and the tension between growing water demand and lessening water availability in developing countries.

## Methods

### Reservoir storage estimation

To estimate the time series of reservoir storage variations, we used the reservoir list/information, the surface area, and the Area-Storage (A-V) relationships.

We extracted the information for 7245 reservoirs from the Global Reservoir and Dam Database (GRanD)^3^ (latest version v1.3). GRanD documents geospatially referenced dams and their associated reservoirs (larger than 0.1 km^3^). GRanD also provides multiple attributes, such as dam height and length, reservoir area, and reservoir storage. The recently released GRanD v1.3 contains 7320 records, with a cumulative reservoir storage capacity of 6811 km^3^. Note that some dams are not associated with reservoirs. We assessed the storage variations for the 7245 reservoirs, which account for a total capacity of 6658 km^3^. There are 57 reservoirs in GRanD without reported storage capacity values. The capacity values for these were estimated based on empirical relationships established in Lehner et al.^3^. However, we found that this did not significantly impact the findings of this study (by comparing the results between including and excluding these reservoirs). Note that this study did not include a comprehensive census of the world’s reservoirs, especially with regard to the very small reservoirs. The GlObal geOreferenced Database of Dams (GOODD) provides the locations of 38,667 dams, but the associated reservoir shapes and other attributes are not available^43^. Lehner et al.^3^ estimate that there were approximately 2000 km^3^ more reservoirs (mainly small reservoirs) than the 6197 km^3^ included in GRanD v1.1 (as of early 2011). Couto and Olden^44^ reveal that 82,891 small hydropower plants (SHPs) are being operated or are under construction—but their analysis is at the nation scale, and georeferenced location information for the individual SHPs are not accessible. Malerba et al.^45^ detected 1.765 million farm dams that occupy a volume of 10.99 km^3^. It has been estimated that the cumulative reservoir storage capacity is around 7000–8300 km^3^ ^1,3,43,46^, and GRanD accounts for about 83% of this.

We adopted the surface area time series values for these reservoirs from the Global Reservoir Surface Area Dataset (GRSAD)^32^. Leveraging the Landsat-based Global Surface Water (GSW) dataset^47^—which contains Earth’s monthly surface water maps at a 30 m resolution from 1984 to 2018—GRSAD corrected the underestimations due to image contamination (e.g., clouds and ice/snow) using a novel algorithm. However, this algorithm cannot be applied to images when more than 95% of the reservoir area is contaminated. Under these conditions, the missing monthly reservoir area values were filled in by a linear interpolation. It should be noted that, although Landsat observations for areas such as the United States and Australia are sufficient for seasonal and interannual evaluations from the early 1980s onward, the coverage for other regions is relatively low before the launch of Landsat-7 in 1999^48–50^. Therefore, the seasonal and interannual variations of reservoir storage were analyzed starting from 1999 in this study.

The monthly storage time series were generated by applying GRSAD area estimations to the A-V relationships for the 7245 reservoirs. The A-V relationships were adopted from two sources. The first is the high-resolution Global Reservoir Bathymetry Dataset (GRBD)^33^, which provides A-V relationships for 347 global artificial reservoirs. By combining the Surface Water Occurrence (SWO) images from GSW with lidar/radar altimetry data, the Area-Elevation (A-E) relationships were first derived for each reservoir. Then, the A-E relationships were, in turn, applied to the corresponding SWO images to obtain the bathymetry values. From the 3-D bathymetric maps, the A-V relationships were then derived^51^.

The other source is a new A-V dataset produced by modifying a simulation method developed by Yigzaw et al.^34^, which was used for the 6898 reservoirs not included in GRBD. The modification was made in two aspects. First, we improved upon the representation of the reservoir areas at capacity. Yigzaw et al.^34^ assumed that the polygon areas in GRanD represented the surface areas at storage capacity. However, the reservoir polygons were primarily based on information from the static Shuttle Radar Topography Mission (SRTM) DEM collected in February of 2000—a period when most reservoirs were unlikely to be at their peaks (because of low fill, a dry season, or some other factors). To minimize this kind of uncertainty, we calculated the 95th percentile area value for each reservoir from the GRSAD dataset, and compared it with the associated area provided in GRanD. The larger value of the two was then selected to represent the area at capacity (for each given reservoir). A total of 5152 reservoir records were updated to the 95th percentile area values. Second, we combined each reservoir’s vertical and bottom profile shapes to estimate the area at each layer by scaling to the area at capacity (i.e., the top layer area). Yigzaw et al.^34^ used the effective length and width of the reservoirs, but did not consider the fact that the complexity of the surface shapes may lead to large uncertainties. We selected three possible bottom profile shapes—parabolic, linear, and square root—and used them in combination with four vertical profile shapes—prism, bowl, wedge, and concave wedge—to obtain twelve different reservoir geometries to choose from. A diagram of a reservoir geometry with a parabolic bottom profile and a prism vertical profile is shown in Supplementary Fig. 3a, with the corresponding parameters denoted in Supplementary Fig. 3b. The selected vertical and bottom profile shapes are shown in Supplementary Figs. 3c and 3d, respectively.

The simulation method included two steps: First, the optimal geometry was determined for each reservoir. The total storage value was calculated for each of the twelve possible geometries by integrating the area with respect to the depth. Then the geometry with the estimated total storage closest to the reported capacity value in GRanD was selected as the optimal geometry. Second, for a given area value, the corresponding storage value was calculated by integration, and then the A-V relationship was derived.

Here we use a reservoir geometry with a parabolic bottom profile and a prism vertical profile (Supplementary Fig. 3a) to demonstrate the process of creating a simulated A-V relationship. As shown in Supplementary Fig. 3b, ${A}_{0}$ represents the area at capacity that is associated with a depth of ${D}_{0}$, which was derived from the dam height $H({D}_{0}=0.95{H})$^34^. It should be noted that for reservoirs with no available dam height information, ${D}_{0}$ was substituted by the average depth—which was calculated using the ratio of storage and area values at capacity. Next, for a given layer with a depth of $D$ which is $z$ meters below the top layer ($z={D}_{0}-D$), its corresponding water area $A$ can be calculated based on the bottom and vertical profile shapes (Eq. (1)).

1 $$A={A}_{0}\sqrt{1-\frac{z}{{D}_{0}}}$$

The estimated total storage $({V}_{0})$ is calculated by integrating the area $(A)$ with respect to the depth (Eq. (2)).

2 $${V}_{0}={\int }_{0}^{{D}_{0}}{A}_{0}\sqrt{1-\frac{z}{{D}_{0}}}\,{dz}=\frac{2}{3}\,{A}_{0}{D}_{0}$$

Similarly, the estimated total storage $({V}_{0})$ values for the other geometries are obtained using the equations in Supplementary Table 3. Then, the reported storage capacity value (${V}_{c}$) is compared with the estimated total storage values, and the geometry with the closest estimation is selected. It should be noted that some geometries have the same estimated storage values, such as a prism vertical profile with a linear bottom profile and a bowl vertical profile with a parabolic bottom profile (Supplementary Table 3). In this case, either one can be selected, which has no effect on the storage calculation.

Next, Eqs. (1) and (2) are combined to derive the A-V relationship (Eq. (3)).

3 $$V=\frac{2}{3}{AD}=\frac{2}{3}A\left({D}_{0}-z\right)=\frac{2}{3}A{D}_{0}{\left(\frac{A}{{A}_{0}}\right)}^{2}$$

The storage equations for other geometries are summarized in Supplementary Table 3.

We selected 16 reservoirs with available in situ A-V relationships from which to compare our modified simulation method with that of Yigzaw et al.^34^ (Supplementary Fig. 4). Results show that our modification can greatly improve the accuracy of the A-V relationships.

### Evaluation of storage estimations

We collected in situ measurements for 277 reservoirs from the United States, Australia, and India to validate the storage results (a total of 101,041 pairs). As shown in Supplementary Fig. 5, the set of estimated storage values agrees well with gauge observations, with an R^2^ value of >0.99 and a slope of 1.01. The volumes of the validation reservoirs are primarily between 2 and 5 km^3^. With regard to the validation results, 49 reservoirs (22 from the United States, 22 from India, and 5 from Australia) employed the A-V relationships derived from bathymetry data. To further evaluate the performance of the two storage estimation methods, we used the simulation method to derive the A-E relationships for these same 49 reservoirs, and then compared the estimated storage values. Supplementary Fig. 6 shows that the estimated storage values from the two methods are in overall good agreement with the observations. For the storage estimations using the bathymetry-based A-V relationships, the coefficient of determination (R^2^) ranges from 0.41 to 0.99, the mean bias error (MBE) from −1.32 km^3^ to 0.63 km^3^, and normalized root mean square error (NRMSE) from 4.40% to 30.80% (Supplementary Table 4). With regard to the results from the simulation-based method, the R^2^, MBE, and NRMSE values range from 0.41 to 0.99, −1.84 km^3^ to 4.92 km^3^, and 6.26% to 127.73%, respectively (Supplementary Table 4). In general, the bathymetry-based method (with mean MBE and NRMSE values of 0.041 km^3^ and 13.28%) performed better than the simulation method (with mean MBE and NRMSE values of 0.23 km^3^ and 24.30%). Although the bathymetry-based A-V relationships are only available for 347 reservoirs, they represent 51.08% of the total global capacity (according to GRanD v1.3)—which can help to reduce the overall error/uncertainty. It should be noted that the storage estimates from the simulation method have a relatively large bias in some cases. For example, the Rana Pratap Sagar, Yeleru, and Nagarjuna Sagar reservoirs have NRMSE values of 127.73%, 87.14%, and 73.09%, respectively. However, the estimated storage values for these reservoirs have good correlations with in situ data (with R^2^ values of 0.80, 0.89, and 0.81, respectively), indicating that the patterns of variation can be successfully captured. Using the Rana Pratap Sagar Reservoir as an example, the long-term trend derived from the simulated storage time series is 0.006 km^3^/yr, which is consistent with the value calculated from in situ measurements (0.005 km^3^/yr). Therefore, the effect of this relatively large bias should not be significant because our analysis is focused on evaluating the trends of storage variation across large scales.

We used the above-mentioned 277 reservoirs to evaluate the uncertainty of the storage dataset. For each month, the total estimated storage value of these reservoirs was compared with the total in situ measurement value to calculate the NRMSE (i.e., 4.15%), which was used to represent the overall uncertainty of the validation dataset. It should be noted that this overall NRMSE value is smaller than the averaged NRMSE value (22.45%) of the 277 individual reservoirs, due to the offset effect between the overestimations and underestimations. In addition, we compared the NS trend values derived from the simulation method with the results from the bathymetry-based methods for the 347 global reservoirs. Supplementary Fig. 7 shows that the NS trend values derived from these two methods show good agreement (R^2^ = 0.76), with the majority of the data aligning with the 1:1 line (with a slope value of 0.99).

### Sources of uncertainty

The storage dataset is associated with uncertainties primarily from two sources. The first is uncertainty due to reservoir sedimentation, which can reduce the storage capacity^52^. Reservoir sedimentation is affected by several major factors, such as geometry, streamflow, sediment load, particle size, deposit-specific weight, reservoir size, and operation rules^53–55^. It has been reported that the sedimentation rate varies with reservoir size, with the larger reservoirs having smaller rates^56–58^. Recently, Wisser et al.^58^ evaluated the storage capacity loss for global reservoirs in GRanD. Their analysis shows that the total storage capacity declined by 4.5% from 1990 to 2010, at an annual rate of 0.23%. Moreover, Dendy et al.^57^ estimated the average annual loss rates for a series of different size categories using the sedimentation data for 1105 reservoirs. Based on the set of loss rates obtained from Dendy et al.^57^, we calculated the annual storage sedimentation values for each reservoir included in the GRS dataset. Then, the total storage sedimentation was divided by the total storage capacity to estimate the annual storage sedimentation rate—0.18%. Currently, a wide range of techniques (e.g., flushing and dredging) have been developed and implemented to control sedimentation, and to ensure the long-term sustainability of the reservoirs^55,59–61^. It should be noted that a fraction of the reservoirs in our dataset (e.g., some in the U.S. and India) were evaluated in terms of live storage, which is more resistant to sedimentation.

Although sedimentation can reduce storage capacity, reservoir management does not allow for water to be stored higher than planned due to concerns about dam safety. One rare exception is when a flood-control reservoir has a recreation pool for which a certain volume of water is required to be maintained, and the surface level (of this required volume) is well below the elevation at capacity. In this case, sedimentation can increase the surface area required to store the specified amount of water. Flushing or dredging is usually applied for mitigating the sedimentation effects on storage. In this study, our method relies on using surface area to infer the storage and does not consider the sedimentation effects. If the sedimentation amount is notable in a reservoir, then both the storage and storage capacity can be overestimated. However, based on the evaluation below, sedimentation has a limited effect on the NS values and the NS trends.

To further evaluate the impacts of sedimentation, we selected 100 U.S. reservoirs that have sediment information from the Duke Nicholas Institute (https://nicholasinstitute.duke.edu/reservoir-national-trends/sediment). The sediment rate values of these reservoirs range from 0 to 18.18%/year, with mean and median values of 1.37%/year and 0.70%/year, respectively (Supplementary Fig. 8a). We compared the NS trend values with and without considering the impacts of sedimentation (Supplementary Fig. 8b). This comparison shows that sedimentation can reduce the NS trend values. Note that the sediment dataset has a relatively large uncertainty due to the scarcity of field measurements, and to the effects of sediment removal activities (e.g., dredging and flushing). As demonstrated in Eqs. (4)–(6), the NS values are lower when considering sedimentation processes, and therefore (while the difference is very minor) the decreasing NS trends presented in this study are conservative.

4 $${{{{{{\rm{NS}}}}}}}=\frac{V}{{V}_{c}}$$

5 $$\,{{{{{{{\rm{NS}}}}}}}}_{{{{{{{\rm{sediment}}}}}}}}=\frac{V-[{{V}_{c}-V}_{c}{\left(1-a\right)}^{t}]}{{V}_{c}{\left(1-a\right)}^{t}}$$

6 $${{{{{{\rm{NS}}}}}}}-{{{{{{{\rm{NS}}}}}}}}_{{{{{{{\rm{sediment}}}}}}}}=\frac{V}{{V}_{c}}-\frac{V-[{{V}_{c}-V}_{c}{\left(1-a\right)}^{t}]}{{V}_{c}{\left(1-a\right)}^{t}}=\frac{\left({V}_{c}-V\right)[1-{(1-a)}^{t}]}{{V}_{c}{(1-a)}^{t}}\; > \;0$$

where $V$ and ${V}_{c}$ are the remotely sensed reservoir storage and storage capacity values (km^3^), $a$ is the sediment rate (%/year), $t$ is the time (year), and ${{{{{{{\rm{NS}}}}}}}}_{{{{{{{\rm{sediment}}}}}}}}$ and ${{{{{{\rm{NS}}}}}}}$ are the normalized storage values with and without considering the sedimentation.

The second source is related to the modified simulation method and the input data. The performance of the mathematical approximation is sometimes not ideal because the reservoir geometry is very complicated. However, it shows that the storage estimations from the simulation method have overall good consistency with in situ measurements (with a mean R^2^ value of 0.81), indicating that they can successfully capture the storage variation patterns. In addition, some uncertainty can be attributed to the input data (i.e., the dam height, and the area and storage values at capacity) provided by GRanD. According to the GRanD technical report, the storage capacity values in GRanD can be classified as “maximum capacity”, “gross capacity”, “normal capacity”, “live capacity” or “minimum capacity”. These are not distinguished in the dataset, which can explain the relatively large vertical bias from the simulation method for the 22 Indian reservoirs (Supplementary Fig. 6 and Supplementary Table 4). The in situ measurements provided by the India Water Resources Information System (India-WRIS) are live storage values. As shown in Supplementary Fig. 9a, the storage capacity values from GRanD are larger than those from India-WRIS. For example, the storage capacity value of Rana Pratap Sagar Reservoir from GRanD (2.90 km^3^) is twice as much as that from India-WRIS (1.436 km^3^)—which resulted a large error (with an NRMSE of 127.73%). Note that the storage values for Indian reservoirs using the bathymetry-based method are live storage values. For the 27 reservoirs in the United States and Australia, the storage capacity values from GRanD agree well with those from water management agencies such as the United States Geological Survey (USGS), the United States Bureau of Recreation (USBR), and the United States Army Corps of Engineers (USACE) (Supplementary Fig. 9b). Therefore, the simulation method performed better in the United States and Australia (with an average NRMSE of 15.38%) than in India (with an average NRMSE of 35.24%). Moreover, this comparison of storage capacity values also indicates that the input data provided by GRanD are reliable.

The water area at capacity is an important input for the simulation model. We adopted the 95-percentile areas to represent the water area at capacity for the majority of the reservoirs. The 100-percentile areas were not selected because these values may correspond to flooding circumstances, which can lead to overestimated water area values at capacity. However, most of the new reservoir polygons added in GRanD v1.3 were based on the maximum (100-percentile) water occurrence extent from the GSW dataset. Therefore, we conducted an uncertainty analysis using the 95- and 100-percentile areas as inputs. Specifically, we used the improved simulation method to derive the storage values from 1999 to 2018, with water-area-at-capacity input values from the 95- and 100-percentile areas, respectively. The in situ storage values of the 277 reservoirs (that we used to validate our storage dataset) were used to validate the simulated storage. Validation results (Supplementary Fig. 10a, b) indicate that the storage values from the 95- and 100-percentile area inputs agree well with the in situ measured values (with R^2^ values of 0.98 and 0.99, and RMSE values of 0.30 km^3^ and 0.29 km^3^). We then compared the storage values derived from these two inputs (Supplementary Fig. 10c), which show good agreement (with a slope of 1.0 and a bias of 0.015 km^3^). In addition, we evaluated the impacts of the water-area-at-capacity inputs on the NS values. We used the 100-percentile areas to replace the 95-percentile areas to generate a different version of the global storage dataset, and then compared the pre-1999 and post-1999 NS values between these two datasets. Results show that the water-area-at-capacity inputs only have a limited impact on the NS values (Supplementary Fig. 10d, e).

### Comparisons with other storage datasets available at a global scale

A number of remotely sensed global reservoir storage datasets have been developed since the early 2010s^27–31^. All of these datasets are based on using the A-E relationships to estimate the reservoir and/or lake storage variations. The main difference between them is the source of the satellite data. For example, Busker et al.^29^ combined the GSW dataset and the satellite altimetry database DAHITI—Database for Hydrological Time Series over Inland Waters—to establish the A-E relationships, which provide long-term storage variation values for 137 lakes and reservoirs. Comparisons of A-E relationships in Busker et al.^29^ with ours show similar quality^33^. Tortini et al.^30^ used MODIS-based water surface areas and a satellite radar altimetry dataset to establish A-E relationships for 347 global lakes and reservoirs. In general, the MODIS-based reservoir storage estimations have larger errors and uncertainties than those of Landsat-based estimations^31^. In addition, the Busker et al.^29^ and Tortini et al.^30^ datasets provide the time series of storage variations (i.e., change in storage) rather than absolute storage values.

### Normalized storage calculation

We used the ratio of the total water storage in all of the reservoirs relative to the total storage capacity for a geographical domain (i.e., global, continental, and river basin scales) to calculate the normalized storage. In this way, the NS can be used as an overall indicator of the relative storage variations at large scales. Note that we did not use the average value of the NS for all of the reservoirs (with each reservoir having equal weight). This is because it is a mathematical average primarily determined by the NS of the large amount small reservoirs (accounting for a small percentage of actual storage), and thus would not represent the actual storage conditions at a large scale. Furthermore, the NS values calculated using the current approach are more physically comparable to the basin scale runoff.

The storage values during the impoundment periods of new reservoirs could impact the NS values. To eliminate this effect, we used two methods to determine the impoundment period for each post-1999 reservoir. For the reservoirs listed in GRanD, we collected their impoundment period information from various sources, such as water management agencies, academic literature, and websites like Wikipedia. Note that public impoundment information is only available for a few reservoirs (e.g., Three Gorges reservoir). For the remainder of the reservoirs, we used a semi-manual method to identify their impoundment periods. We plotted their storage time series and then identified the turning point after which the storage values maintained consistent seasonal variations. For each of the post-1999 reservoirs, the storage values during their impoundment periods were removed before they were used for calculating the NS time series.

### Comparison of NS values between pre- and post-1999 reservoirs

The operation of a reservoir depends on the primary reservoir purpose, which can impact the NS level. We compared the NS values for global pre- and post-1999 reservoirs in terms of reservoir function. Figure 2a shows that post-1999 reservoirs tend to be designed for hydropower purposes (vs. pre-1999 ones; 80.9% vs. 63.6%), but less for irrigation (7.8% vs. 19.9%) and flood control (3.4% vs. 6.9%). The NS values for post-1999 reservoirs are lower (with larger seasonal variations) than pre-1999 reservoirs (Fig. 2b–d) for all of the reservoir functions—hydropower (63.65 ± 4.66% vs. 71.37 ± 1.84%), irrigation (52.39 ± 8.53% vs. 60.83 ± 2.88%), and flood control (50.98 ± 6.71% vs. 56.90 ± 3.09%). At the continental scale, the locations of pre- and post-1999 reservoirs may play a role, because of the heterogeneous climatology within the continent. Thus, we conducted the comparison—taking the reservoir function into consideration—at the basin scale, assuming that the climatology conditions within a basin were homogeneous. We only selected basins that have at least five post-1999 reservoirs with the same primary function, such that the NS values would not be biased by a small number of samples. A total of ten basins were identified that met this criterion. Results show that, in nine out of the ten basins studied, the mean NS values of post-1999 reservoirs are lower than those of pre-1999 reservoirs—with the exception of post-1999 reservoirs used for irrigation purposes in the India-South basin (Fig. 2e and Supplementary Table 1). With regard to mean annual CV values, the pre- and post-1999 reservoirs used for irrigation in the same basins have similar seasonal variations (Supplementary Table 1). However, post-1999 reservoirs with hydropower as their primary purpose exhibit greater seasonal variation than pre-1999 (hydropower focused) reservoirs—except for those in the Parana basin (Supplementary Table 1). Thus, these comparisons indicate that post-1999 reservoirs have lower NS levels—but larger seasonal variations—than pre-1999 ones.

### Trend analysis of storage and NS time series

We adopted the scheme developed by Pascolini-Campbell et al.^62^ to evaluate the trends of storage and NS time series. First, the seasonal cycle was removed using the climatology values, and then a moving average of 15 months was applied to obtain the interannual variability of the anomaly time series. Then, the non-parametric Mann-Kendall significance test^63,64^ was used to statistically assess if there was a monotonic upward or downward trend of storage (or NS) over time (with α = 0.05). Finally, the Theil-Sen slope estimator^65,66^ was employed to detect the linear trend of storage (or NS) time series. Note that the first and last seven monthly values of the time series were not used for the trend analysis.

### Analysis of potential drivers of NS patterns

We analyzed the potential drivers of the NS patterns at the basin scale, which included runoff, population density, and reservoir use. The runoff data were provided by the GLDAS Noah Land Surface Model L4 monthly 0.25 × 0.25 degree V2.1 (GLDAS_NOAH025_M 2.1) product from 2000 to 2018^67^. We calculated the long-term mean runoff value for each basin that contained the studied reservoirs. The gridded population density data were collected from the Gridded Population of the World, Version 4 (GPWv4) data sets—which provide an estimate of population density for the years 2000, 2005, 2010, 2015, and 2020 (https://sedac.ciesin.columbia.edu/data/set/gpw-v4-population-density-adjusted-to-2015-unwpp-country-totals-rev11). We used the average value from these five years to represent the population density for each basin. The primary function of reservoirs is dominated by hydropower across the world (Fig. 2a), and the NS values of hydropower reservoirs are generally higher than those of others (Fig. 2b–d). We calculated the storage capacity percentage of hydropower reservoirs for each basin. Then, we analyzed the correlation between these three drivers and the long-term mean NS values (2000–2018) at the basin scale (Fig. 5). Note that a log-log transformation was applied for the evaluation of the runoff and population density to correct for heteroskedasticity.

Runoff varies greatly across global basins, with low values in Central Asia, southern Africa, and Oceania; and high values in the tropical regions and South Asia (Fig. 5a). As shown in Fig. 5b, the long-term mean runoff and NS values show a significant positive correlation (Pearson correlation r = 0.21, *p* < 0.05). The East and South Asia regions are the most densely populated, represented by China and India (Fig. 5c). Conversely, the high-latitude regions, the Amazon, and Oceania are each sparsely populated (Fig. 5c). As indicated in Fig. 5d, population density is significantly correlated with the NS pattern (r = −0.36, *p* < 0.001). We used the storage capacity percentage of hydropower reservoirs for evaluating the role of reservoir function, as hydropower reservoirs need to maintain relatively high water levels to ensure that their generators operate efficiently. Figure 5e shows the storage capacity percentage of the hydropower reservoirs within each basin. The reservoirs in the high-latitude basins are primarily designed for hydropower (and therefore have high hydropower percentages), while the basins in sub-tropical regions have relatively low hydropower percentages. However, the basins in South America and central Africa also have high hydropower percentages. The significantly positive correlation (Fig. 5f) between hydropower percentage and NS (r = 0.37, *p* < 0.001) indicates that reservoir function is also an important driver of NS level.

## Supplementary information

Supplementary Information

Peer Review File

## Data Availability

The Global Reservoir Storage (GRS) dataset generated in this study has been deposited in the Zenodo^68^ under accession code 10.5281/zenodo.7855477, with an interactive map on Google Earth Engine platform (https://yao.users.earthengine.app/view/grs). The in situ measurements for validating the storage estimations were provided by the United States Geological Survey (USGS), the United States Bureau of Recreation (USBR), the United States Army Corps of Engineers (USACE), the California Data Exchange Center (CDEC), the Australia Bureau of Meteorology (BOM), and the India Water Resources Information System (India-WRIS).

## Code availability

The codes used for computations and statistical analyses are available at 10.5281/zenodo.7874111.
