# Supplementary material for: Diminishing storage returns of reservoir construction
Source: Nat Commun. 2023 Jun 13;14:3203. doi: 10.1038/s41467-023-38843-5 (PMC10264411; doi:10.1038/s41467-023-38843-5)
Supplement: Supplementary file 1 — Supplementary Information [file 41467_2023_38843_MOESM1_ESM.pdf]

Supplementary Information for

Diminishing Storage Returns of Reservoir Construction

Yao Li[1#], Gang Zhao[2], George H. Allen[3], and Huilin Gao[1*]

[1]Zachry Department of Civil and Environmental Engineering, Texas A&M University, College Station, TX, USA

[2]Department of Global Ecology, Carnegie Institution for Science, Stanford, CA, USA

[3]Department of Geosciences, Virginia Polytechnic and State University, Blacksburg, VA, USA

[#]Current affiliation: Chongqing Jinfo Mountain Karst Ecosystem National Observation and Research Station, School of Geographical Sciences, Southwest University, Chongqing, China

[*]Corresponding author: hgao@civil.tamu.edu

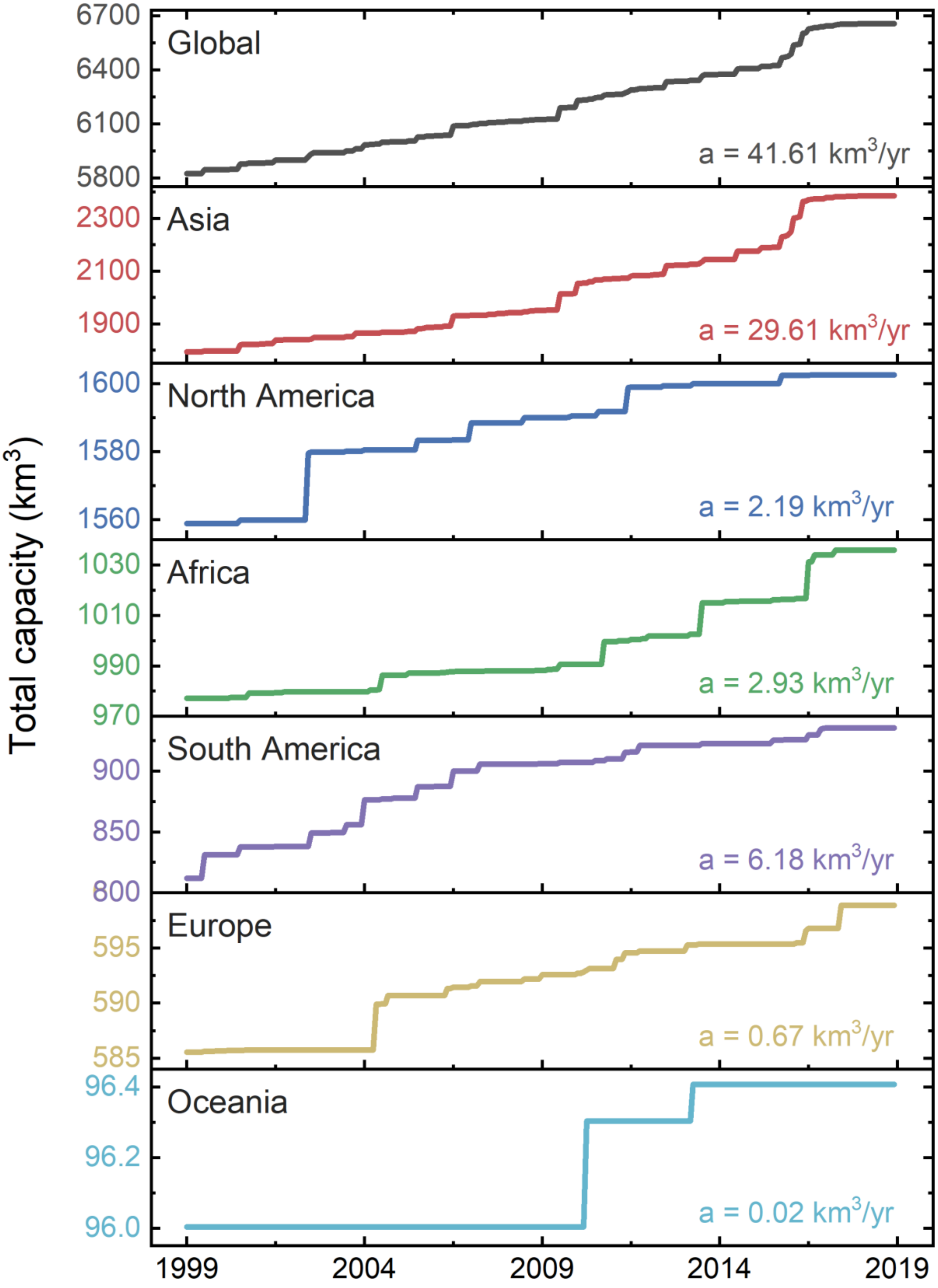

Supplementary Figure 1. Global and continental total reservoir capacity values from 1999 to 2018.

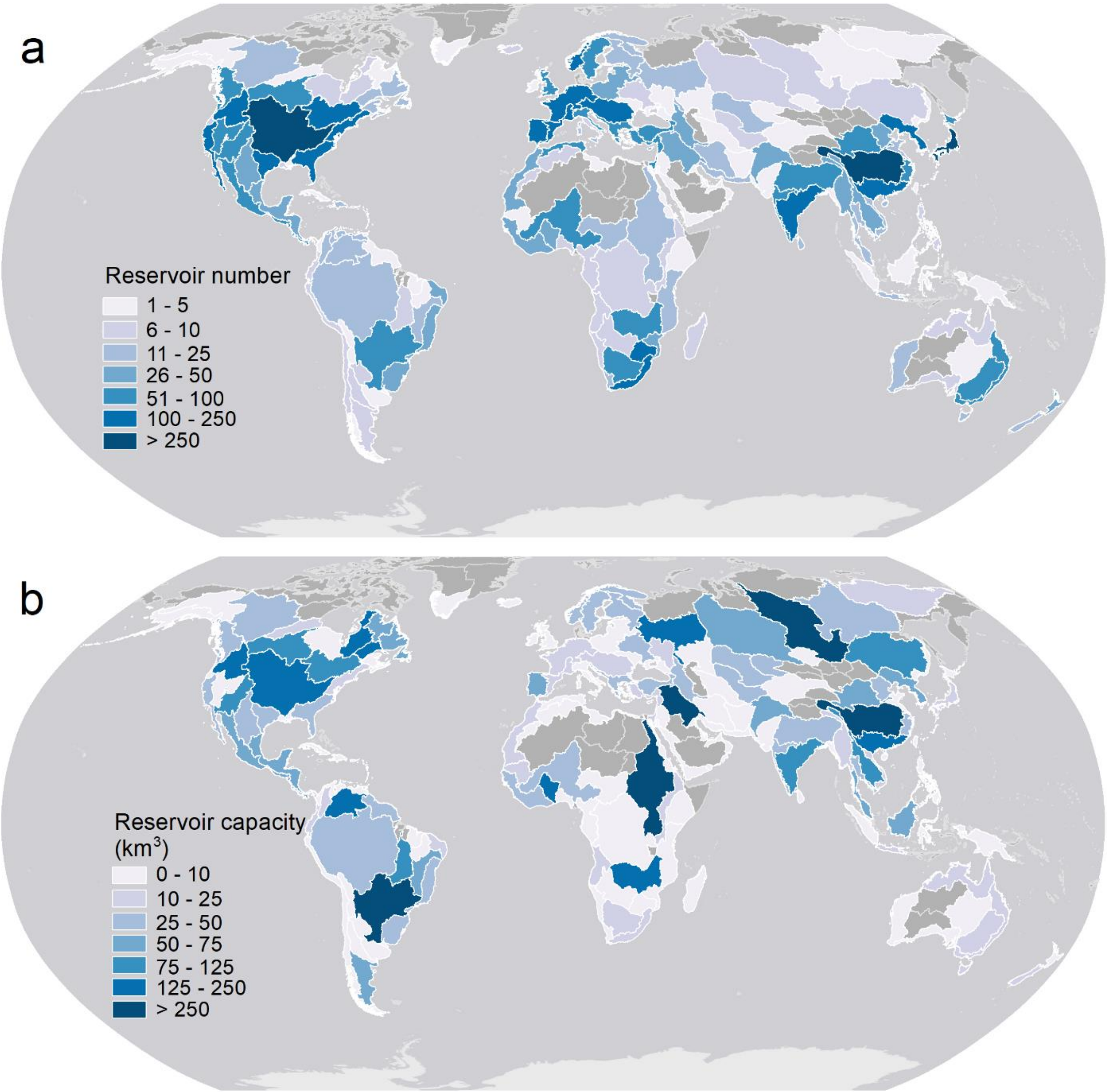

Supplementary Figure 2. Reservoir (a) number and (b) total capacity at the basin scale.

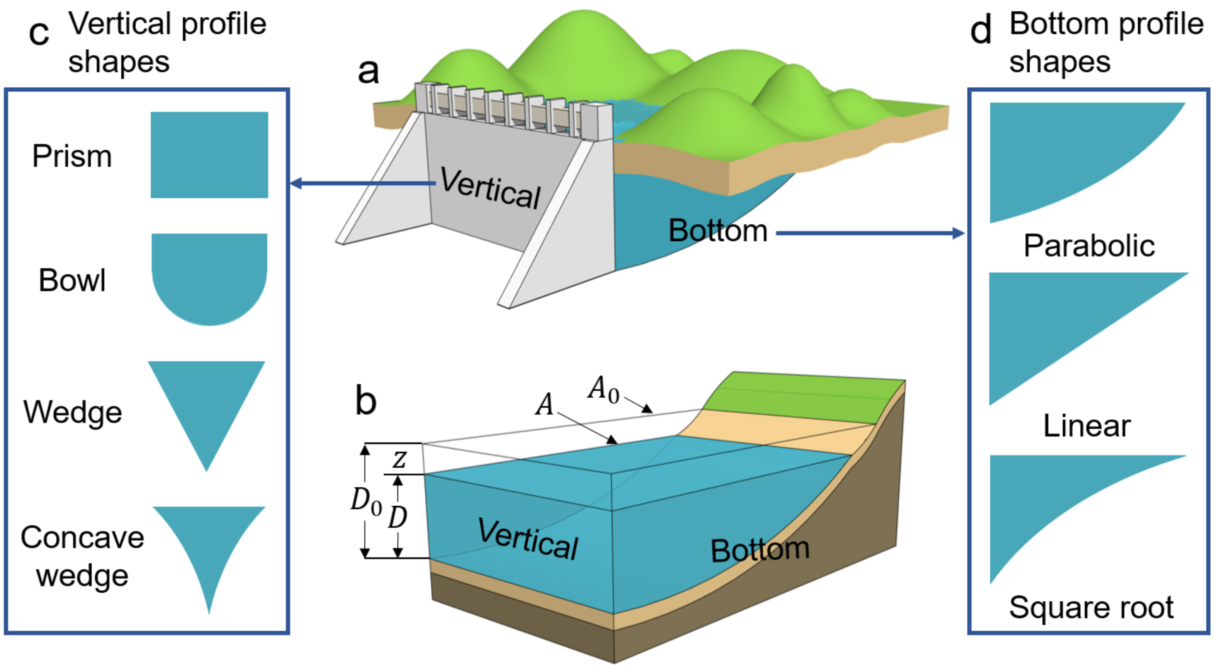

Supplementary Figure 3. (a) A diagram of a reservoir geometry that consists of a parabolic bottom profile and a prism vertical profile—with the corresponding parameters denoted in (b). All of the selected bottom and vertical profile shapes are shown in (c) and (d), respectively. A_0 represents the area at capacity that is associated with a depth of D_0. For a given layer with an area of A, its depth corresponds to D, and $z = D_0 - D$ (which is the vertical distance between the layers A and A_0).

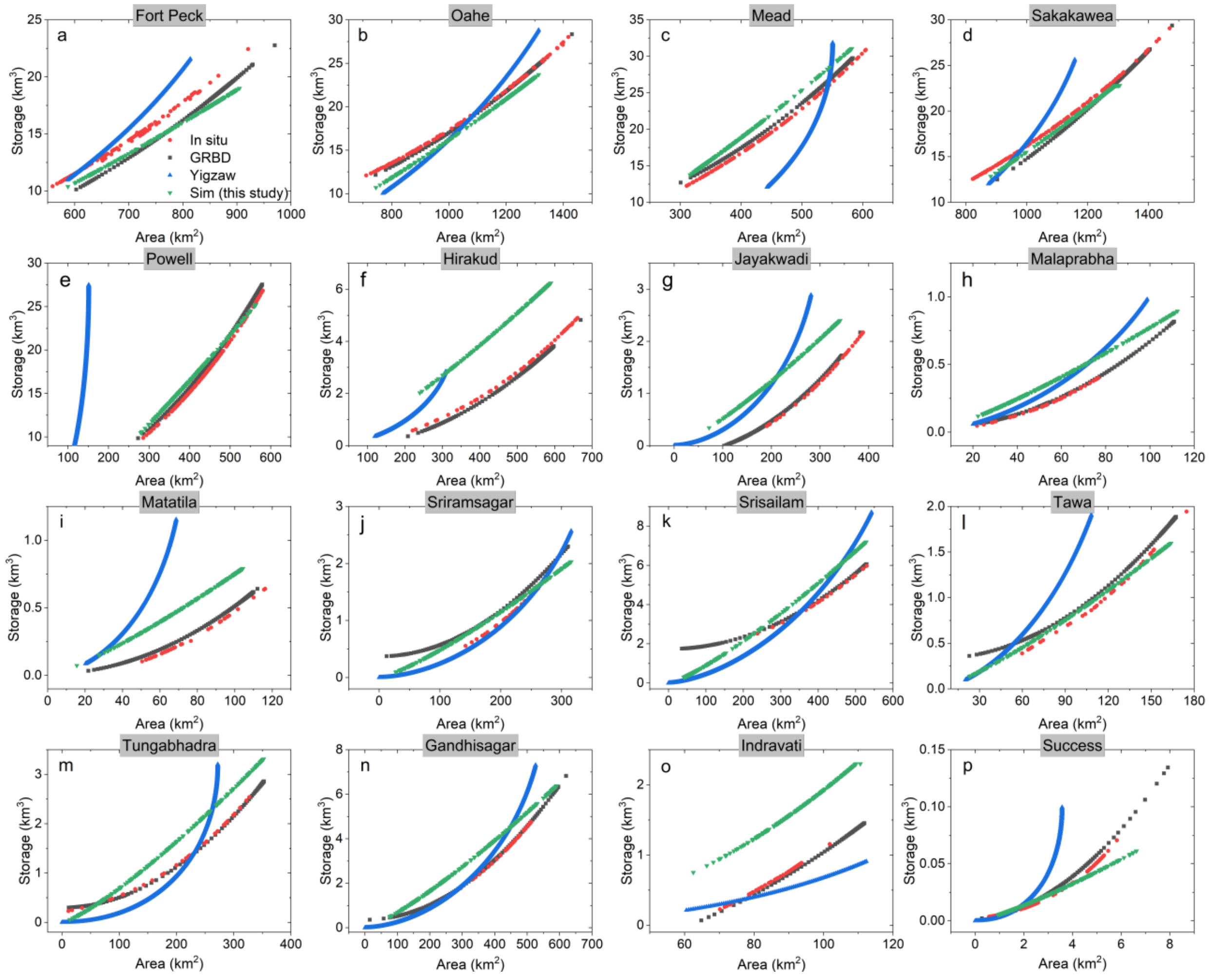

Supplementary Figure 4. Comparison of the Area-Storage (A-V) relationships derived from the Global Reservoir Bathymetry Dataset (GRBD), Yigzaw, et al.[1] and the modified simulation method (this study) over the (a) Fort Peck, (b) Oahe, (c) Mead, (d) Sakakawea, (e) Powell, (f) Hirakud, (g) Jayakwadi, (h) Malaprabha, (i) Matatila, (j) Sriramsagar, (k) Srisailam, (l) Tawa, (m) Tungabhadra, (n) Gandhisagar, (o) Indravati, and (p) Success reservoirs.

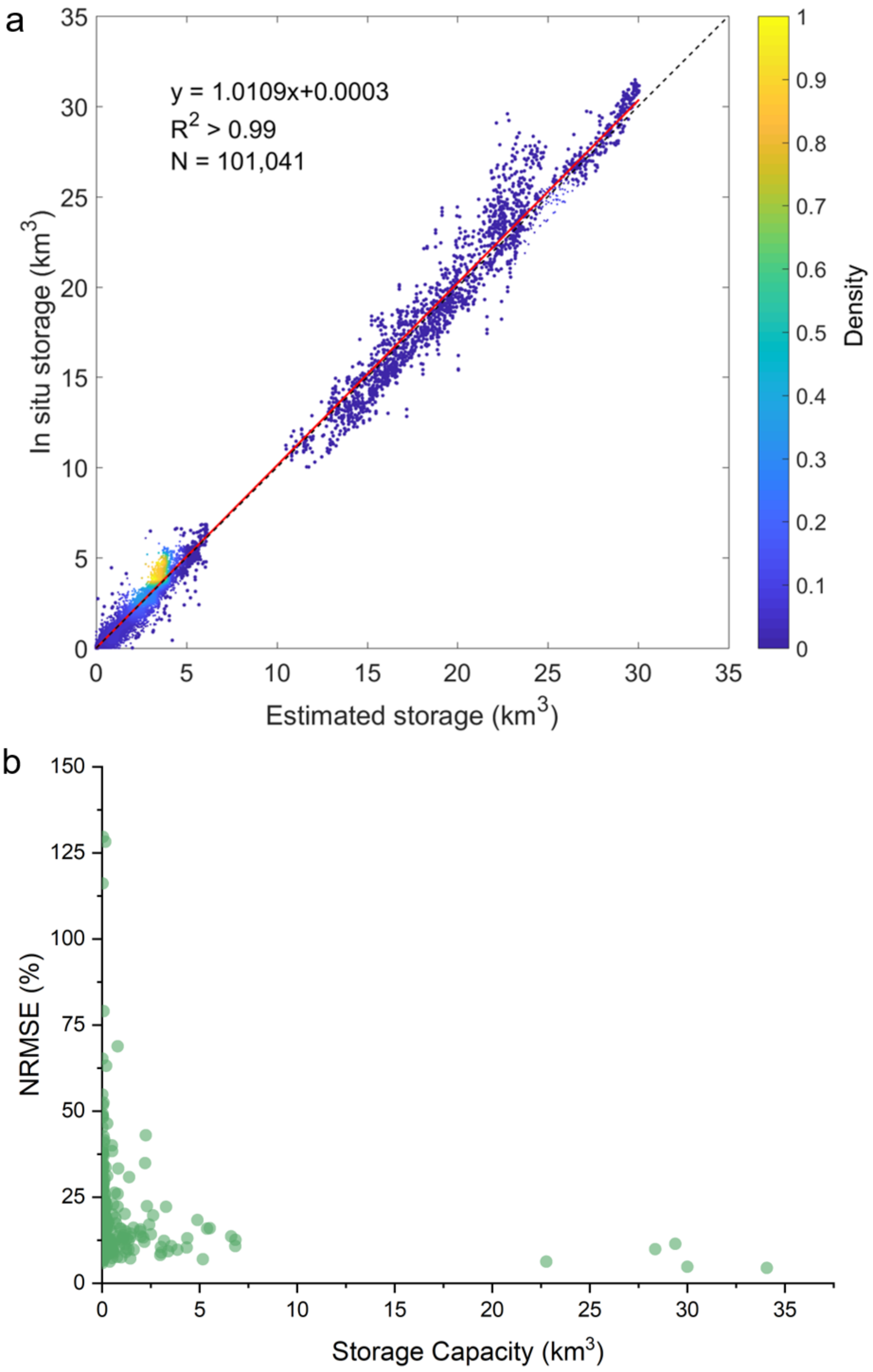

Supplementary Figure 5. (a) Comparison of the estimated storage values with *in situ* measurements for 277 reservoirs in the United States, Australia, and India; (b) Scatter plot of normalized root mean square error (NRMSE) values and the storage capacity values for these 277 validation reservoirs.

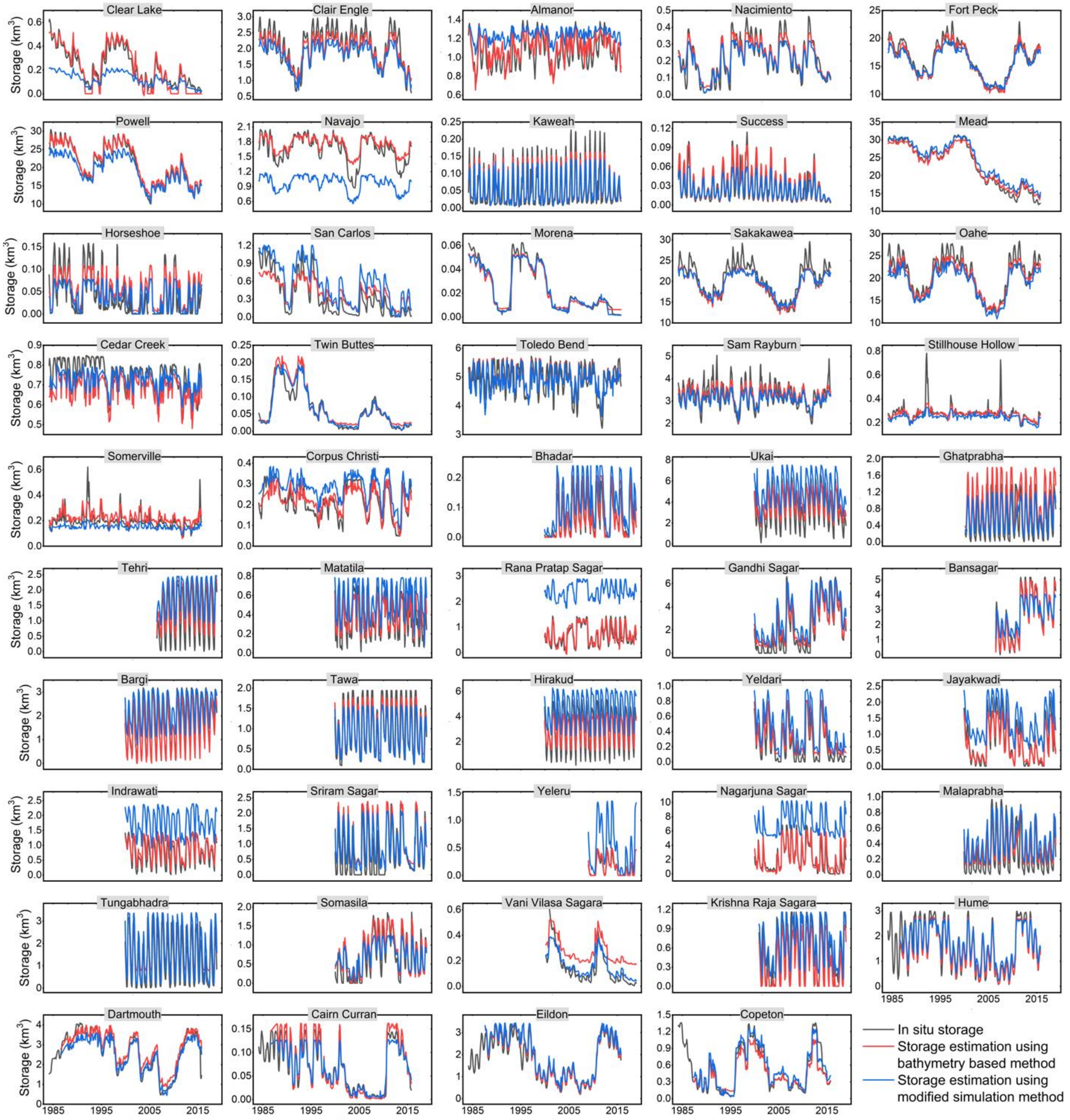

Supplementary Figure 6. Validation of storage estimations using bathymetry based and simulation based methods against *in situ* values for 49 reservoirs in the United States (22), India (22), and Australia (5).

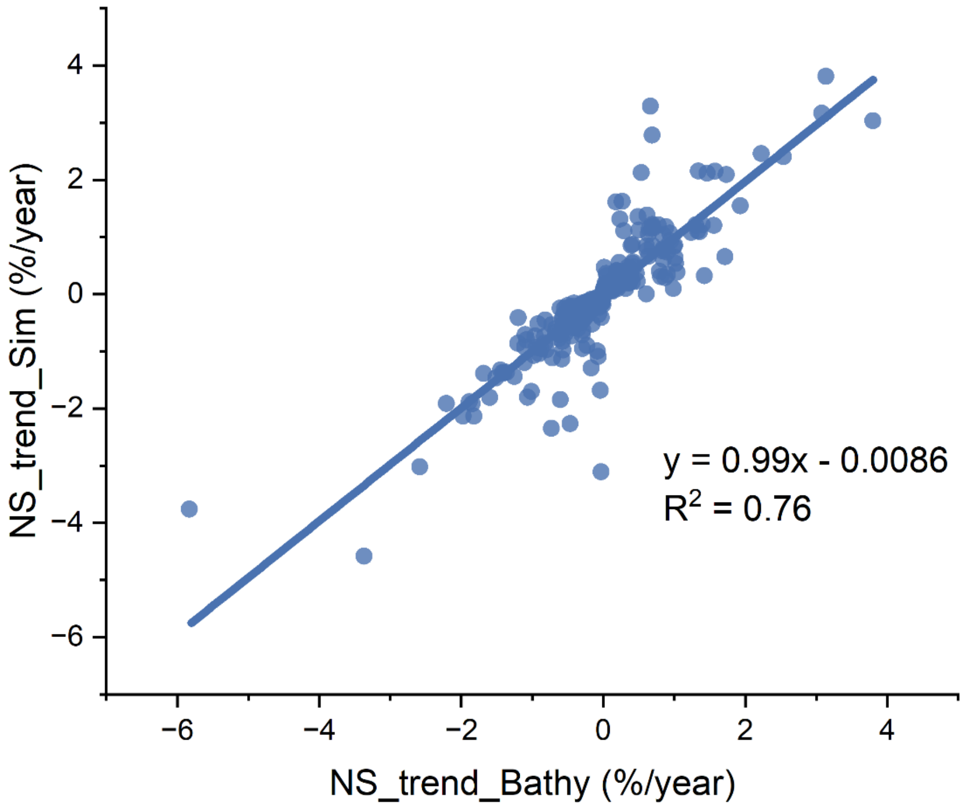

Supplementary Figure 7. Comparison of NS trend values derived from bathymetry and simulation based methods for the 347 global reservoirs.

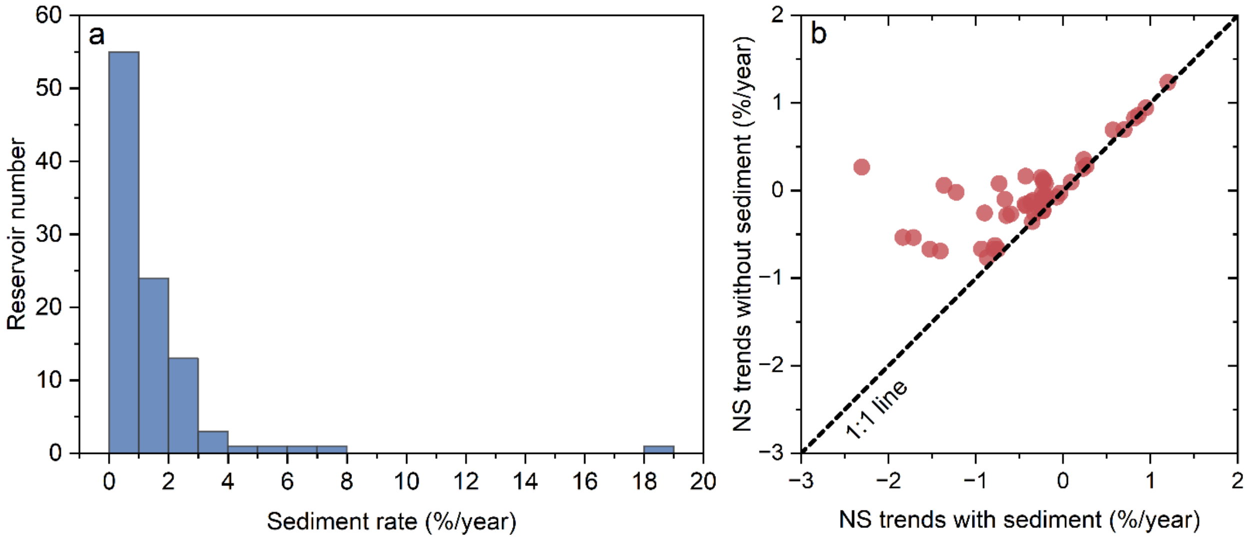

Supplementary Figure 8. (a) Distribution of the sediment rates for the 100 reservoirs used to evaluate the effects of sedimentation on the NS trends; (b) Comparison of NS trend values with and without considering sedimentation.

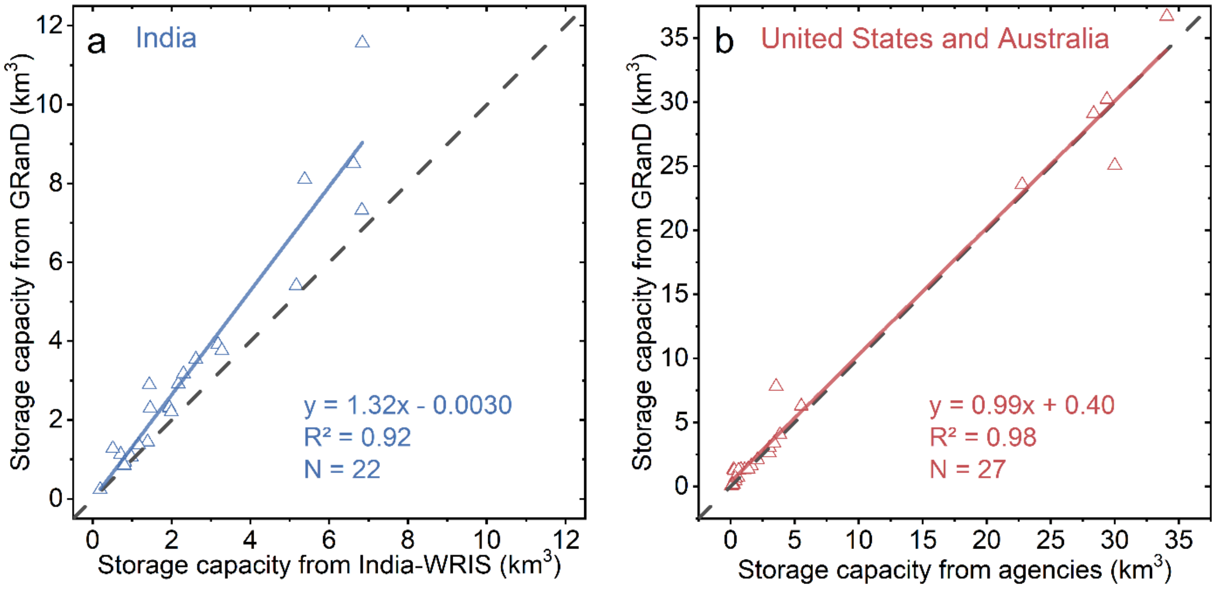

Supplementary Figure 9. Comparison of storage capacity values from water management agencies and the Global Reservoir and Dam Database (GRanD) for the 49 reservoirs (included in Supplementary Figure 6) in (a) India, and (b) the United States and Australia.

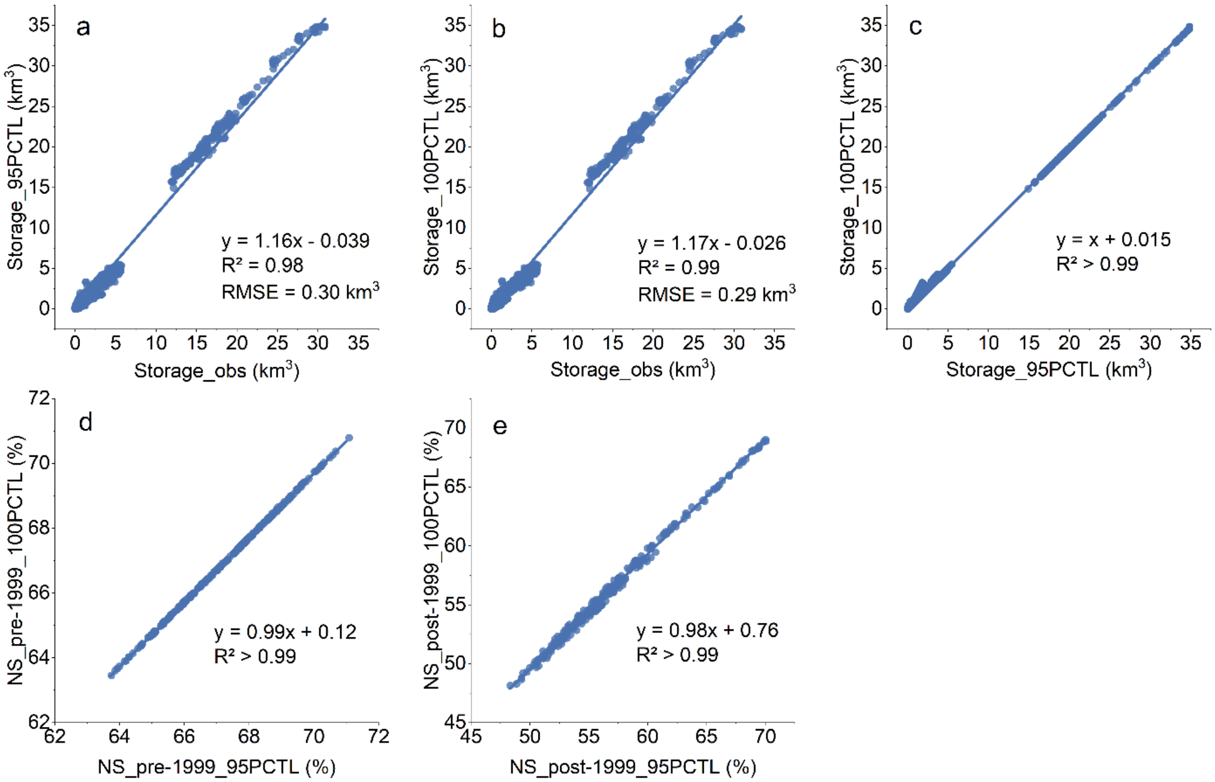

Supplementary Figure 10. (a-b) Validation of simulated storage values from 1999 to 2018 with water area at capacity values from 95- and 100-percentile areas (respectively) used as inputs; (c) Comparison of the simulated storage values derived from these two inputs; (d-e) Comparison of NS values for pre- and post-1999 reservoirs between global storage datasets using the 95- and 100-percentile areas as water-area-at-capacity inputs.

Supplementary Table 1. Statistics of the normalized storage (NS) comparison between pre- and post-1999 reservoirs at the basin scale.

Basin	PF	Post-reservoirs				Pre-1999 reservoirs			
		#	TC (km^3)	Mean NS (%)	Mean annual CV (%)	#	TC (km^3)	Mean NS (%)	Mean annual CV (%)
Turkey-MED	IR	9	2.98	44.48 ± 7.82	9.95	5	5.52	53.77 ± 11.15	11.06
Turkey-BLK	HP	9	7.49	64.48 ± 5.80	7.94	5	14.78	71.37 ± 2.50	2.47
Euphrates	HP	15	23.68	43.92 ± 8.52	6.90	3	41.18	62.50 ± 3.56	3.44
Yangtze	HP	69	137.92	53.73 ± 10.76	20.02	45	63.83	56.40 ± 8.62	15.29
China-SE	HP	6	7.01	50.95 ± 13.32	19.00	9	28.74	52.32 ± 4.75	8.10
Pearl	HP	33	65.75	51.01 ± 14.18	24.33	19	26.76	58.01 ± 7.57	10.81
Mekong	HP	16	57.32	67.21 ± 9.25	12.60	6	9.60	76.20 ± 7.71	9.80
India-South	IR	7	4.19	59.30 ± 19.29	31.47	115	55.91	47.55 ± 15.43	28.68
India-WC	IR	5	2.79	51.41 ± 15.64	22.99	32	17.92	61.22 ± 14.80	23.05
Parana	HP	10	33.90	61.97 ± 1.85	2.38	31	238.62	69.50 ± 7.56	5.89

The reservoirs with the primary function (PF) of irrigation (IR) and hydropower (HP) were evaluated. For each basin, # and TC represent the number and total capacity of the reservoirs, respectively. Mean NS values were derived from the monthly NS time series, with the uncertainties represented by standard deviations. The annual coefficient of variation (CV) is defined as the standard deviation of the NS divided by the mean value, which is based on the 12 monthly values from each year. The mean NS values were used to evaluate the overall NS levels, and the mean annual CV values were adopted to assess the seasonal variations.

Supplementary Table 2. Statistics of global and continental storage variations from 1999 to 2018

	Globe	Asia	North America	Africa	South America	Europe	Oceania
Reservoir number	7,245	2,352	2,284	752	341	1,265	251
Total capacity (km^3)	6,657.58	2,386.78	1,602.57	1,037.02	935.92	598.88	96.41
Average capacity (km^3)	0.92	1.01	0.70	1.38	2.74	0.47	0.38
Mean storage (km^3)	4,236.32 ± 181.64	1,275.30 ± 136.18	1,156.93 ± 34.87	736.18 ± 32.38	593.00 ± 48.89	418.75 ± 10.70	56.16 ± 6.49
Mean normalized storage (NS, %)	68.44 ±1.41	62.98 ±2.98	72.90 ±2.00	73.83 ±3.41	66.37 ±4.83	70.75 ±1.63	58.39 ±6.70
Mean annual CV of NS (%)	1.47	4.38	1.49	2.29	4.09	1.98	2.91
Storage trend (km^3/yr)	27.82 ± 0.076 ***	16.76 ± 0.049 ***	3.82 ± 0.015 ***	0.93 ± 0.0092 ***	2.13 ± 0.020 ***	0.97 ± 0.0025 ***	0.024 ± 0.0027
NS trend (%/yr)	-0.041 ± 0.00026 **	-0.0092 ± 0.00037	0.13 ± 0.00065 ***	-0.20 ± 0.0015 ***	-0.18 ± 0.0019 **	0.075 ± 0.00028 ***	0.0073 ± 0.0026
Pre-1999 reservoirs							
Reservoir number	6,728	1,987	2,260	714	279	1,239	249
Total capacity (km^3)	5,822.80	1,793.58	1,558.76	977.14	811.76	585.55	96.00
Average capacity (km^3)	0.87	0.90	0.69	1.37	2.91	0.47	0.39
Mean storage (km^3)	4,009.24 ± 80.05	1,145.52 ± 48.86	1,134.94 ± 32.00	722.24 ± 33.02	535.35 ± 42.69	415.17 ± 9.70	56.03 ± 6.44
Mean NS (%)	68.85 ±1.37	63.87 ±2.72	72.81 ±2.05	73.91 ±3.38	65.95 ±5.26	70.90 ±1.66	58.36 ±6.71
Storage trend (km^3/yr)	-0.048 ± 0.016	1.16 ± 0.0075 ***	2.05 ± 0.011 ***	-1.86 ± 0.015 ***	-1.74 ± 0.019 ***	0.54 ± 0.0016 ***	-0.0015 ± 0.0027
NS trend (%/yr)	-0.00081 ± 0.00026	0.065 ± 0.00037 ***	0.13 ± 0.00069 ***	-0.19 ± 0.0015 ***	-0.21 ± 0.0022 ***	0.092 ± 0.00027 ***	-0.0017 ± 0.0027

*, **, and *** represent $0.01 < p < 0.05$, $0.001 < p < 0.01$, and $p < 0.001$, respectively. All uncertainties are standard deviations. Mean storage and normalized storage (NS) were derived from the monthly observations from 1999 to 2018. The annual coefficient of variation (CV) is defined as the standard deviation of the NS divided by the mean value, which is based on the 12 monthly values from each year. The mean NS values were used to evaluate the overall NS levels, and the mean annual CV values were adopted to assess the seasonal variations.

Supplementary Table 3. Summary of simulated area and storage equations for each geometry

	Parabolic	Linear	Square root
Prism	$A_i = A_0\sqrt{1-\frac{z}{D_0}}$	$A_i = A_0\,(1-\frac{z}{D_0})$	$A_i = A_0\,(1-\frac{z}{D_0})^2$
	$V_0 = \int_0^{D_0} A_i\,dz = \frac{2}{3}\,A_0 D_0$	$V_0 = \int_0^{D_0} A_i\,dz = \frac{1}{2}\,A_0\,D_0$	$V_0 = \int_0^{D_0} A_i\,dz = \frac{1}{3}\,A_0\,D_0$
	$V = \frac{2}{3}AD = \frac{2}{3}AD_0(\frac{A}{A_0})^2$	$V = \frac{1}{2}AD = \frac{1}{2}AD_0\frac{A}{A_0}$	$V = \frac{1}{3}AD = \frac{1}{3}AD_0(\frac{A}{A_0})^{\frac{1}{2}}$
Bowl	$A_i = A_0\sqrt{1-\frac{z}{D_0}}\sqrt{1-\frac{z}{D_0}}$	$A_i = A_0(1-\frac{z}{D_0})\sqrt{1-\frac{z}{D_0}}$	$A_i = A_0\,(1-\frac{z}{D_0})^2\sqrt{1-\frac{z}{D_0}}$
	$V_0 = \int_0^{D_0} A_i\,dz = \frac{1}{2}\,A_0\,D_0$	$V_0 = \int_0^{D_0} A_i\,dz = \frac{2}{5}\,A_0\,D_0$	$V_0 = \int_0^{D_0} A_i\,dz = \frac{2}{7}\,A_0\,D_0$
	$V = \frac{1}{2}AD = \frac{1}{2}AD_0\frac{A}{A_0}$	$V = \frac{2}{5}AD = \frac{2}{5}AD_0(\frac{A}{A_0})^{\frac{2}{3}}$	$V = \frac{2}{7}AD = \frac{2}{7}AD_0(\frac{A}{A_0})^{\frac{2}{5}}$
Wedge	$A_i = A_0\sqrt{1-\frac{z}{D_0}}\,(1-\frac{z}{D_0})$	$A_i = A_0\,(1-\frac{z}{D_0})(1-\frac{z}{D_0})$	$A_i = A_0\,(1-\frac{z}{D_0})^2(1-\frac{z}{D_0})$
	$V_0 = \int_0^{D_0} A_i\,dz = \frac{2}{5}\,A_0\,D_0$	$V_0 = \int_0^{D_0} A_i\,dz = \frac{1}{3}\,A_0\,D_0$	$V_0 = \int_0^{D_0} A_i\,dz = \frac{1}{4}\,A_0\,D_0$
	$V = \frac{2}{5}AD = \frac{2}{5}AD_0(\frac{A}{A_0})^{\frac{2}{3}}$	$V = \frac{1}{3}AD = \frac{1}{3}AD_0(\frac{A}{A_0})^{\frac{1}{2}}$	$V = \frac{1}{4}AD = \frac{1}{4}AD_0(\frac{A}{A_0})^{\frac{1}{3}}$
Concave wedge	$A_i = A_0\sqrt{1-\frac{z}{D_0}}\,(1-\frac{z}{D_0})^2$	$A_i = A_0\,(1-\frac{z}{D_0})\,(1-\frac{z}{D_0})^2$	$A_i = A_0\,(1-\frac{z}{D_0})^2\,(1-\frac{z}{D_0})^2$
	$V_0 = \int_0^{D_0} A_i\,dz = \frac{2}{7}\,A_0\,D_0$	$V_0 = \int_0^{D_0} A_i\,dz = \frac{1}{4}\,A_0\,D_0$	$V_0 = \int_0^{D_0} A_i\,dz = \frac{1}{5}\,A_0\,D_0$
	$V = \frac{2}{7}AD = \frac{2}{7}AD_0(\frac{A}{A_0})^{\frac{2}{5}}$	$V = \frac{1}{4}AD = \frac{1}{4}AD_0(\frac{A}{A_0})^{\frac{1}{3}}$	$V = \frac{1}{5}AD = \frac{1}{5}AD_0(\frac{A}{A_0})^{\frac{1}{4}}$

A_0 represents the area at capacity that is associated with a depth of D_0. For a given layer with an area of A, its depth corresponds to D, and $z = D_0 - D$ (which is the vertical distance between the layers A and A_0).

Supplementary Table 4. Summary of the validation results.

GRanD ID	Reservoir name	Ctry	[a]Cap. (km^3)	R^2		MBE (km^3)		NRMSE	
				[b]Bathy.	[c]SIM.	Bathy.	SIM.	Bathy.	SIM.
119	Clear Lake	U.S.	0.56	0.93	0.90	-0.0052	-0.073	9.17%	23.42%
131	Clair Engle	U.S.	3.02	0.89	0.88	-0.087	-0.27	10.48%	15.52%
138	Almanor	U.S.	1.61	0.61	0.61	0.046	0.18	16.10%	32.44%
231	Nacimiento	U.S.	0.47	0.89	0.87	0.0070	-0.015	9.34%	11.23%
307	Fort Peck	U.S.	22.77	0.96	0.96	0.63	-0.68	6.27%	8.67%
597	Powell	U.S.	30.00	0.98	0.98	0.53	-1.60	4.74%	10.86%
601	Navajo	U.S.	2.11	0.94	0.95	0.097	-0.68	13.25%	58.65%
605	Kaweah	U.S.	0.23	0.73	0.71	0.0063	0.0034	13.25%	13.60%
609	Success	U.S.	0.10	0.82	0.80	0.0036	-0.0020	9.72%	10.76%
610	Mead	U.S.	34.07	0.99	0.99	0.094	1.40	4.40%	8.34%
640	Horseshoe	U.S.	0.13	0.73	0.68	0.014	-0.0002	17.74%	17.79%
656	San Carlos	U.S.	1.12	0.90	0.90	0.067	0.24	13.86%	22.23%
669	Morena	U.S.	0.062	0.98	0.97	-0.0012	-0.0011	6.54%	6.26%
753	Sakakawea	U.S.	29.38	0.90	0.89	-1.32	-1.05	11.43%	11.33%
870	Oahe	U.S.	28.35	0.92	0.92	-0.86	-1.84	9.87%	14.09%
1230	Cedar Creek	U.S.	0.80	0.44	0.44	-0.062	-0.022	25.90%	18.77%
1263	Twin Buttes	U.S.	0.23	0.91	0.93	0.0069	0.0019	10.36%	7.66%
1269	Toledo Bend	U.S.	5.52	0.41	0.41	0.0049	-0.023	15.94%	15.83%
1275	Sam Rayburn	U.S.	3.55	0.65	0.65	-0.026	-0.17	10.75%	12.79%
1277	Stillhouse Hollow	U.S.	0.28	0.57	0.53	-0.011	-0.036	8.21%	10.09%
1296	Somerville	U.S.	0.62	0.61	0.57	0.029	-0.046	8.79%	12.17%
1317	Corpus Christi	U.S.	0.32	0.81	0.79	0.0041	0.066	12.55%	26.22%
4735	Bhadar	India	0.19	0.78	0.78	0.0076	0.050	18.78%	33.63%
4739	Ukai	India	6.62	0.86	0.86	0.51	1.91	13.60%	31.50%
4773	Ghatprabha	India	1.39	0.67	0.68	0.31	0.042	30.80%	20.34%
4795	Tehri	India	2.62	0.79	0.78	0.29	0.58	19.67%	29.29%
4826	Matatila	India	0.71	0.68	0.67	0.030	0.18	17.39%	31.32%
4836	Rana Pratap Sagar	India	1.44	0.80	0.80	0.058	1.74	13.32%	127.73%
4843	Gandhi Sagar	India	6.83	0.92	0.91	0.35	0.94	10.74%	17.32%
4859	Bansagar	India	5.17	0.95	0.95	-0.051	0.071	6.96%	12.10%
4881	Bargi	India	3.18	0.85	0.84	-0.021	0.64	12.29%	25.25%
4885	Tawa	India	1.94	0.82	0.82	-0.0074	-0.11	15.00%	16.51%
4898	Hirakud	India	5.38	0.80	0.79	0.0037	1.76	15.82%	36.46%
4938	Yeldari	India	0.82	0.90	0.88	0.078	0.20	13.57%	27.05%
4942	Jayakwadi	India	2.17	0.87	0.87	-0.074	0.65	12.01%	31.85%
4943	Indrawati	India	1.46	0.84	0.84	0.13	1.01	14.38%	70.76%
4946	Sriram Sagar	India	2.30	0.73	0.71	0.31	0.23	22.36%	21.10%
4978	Yeleru	India	0.51	0.90	0.89	-0.00046	0.35	10.38%	87.14%

4985	Nagarjuna Sagar	India	6.84	0.82	0.81	0.13	4.92	12.58%	73.09%
4992	Malaprabha	India	0.97	0.76	0.75	0.08	0.15	15.83%	20.53%
4994	Tungabhadra	India	3.28	0.75	0.77	0.48	0.36	22.16%	19.86%
4997	Somasila	India	1.99	0.82	0.80	0.12	0.022	13.64%	14.91%
5000	Vani Vilasa Sagara	India	0.80	0.91	0.89	0.13	0.021	22.19%	9.00%
5009	Krishna Raja Sagara	India	1.16	0.82	0.81	-0.16	0.13	20.13%	18.61%
6628	Hume	AU.	3.04	0.93	0.92	-0.063	0.079	8.87%	10.44%
6637	Dartmouth	AU.	3.86	0.93	0.93	0.18	-0.14	9.71%	9.34%
6647	Cairn Curran	AU.	0.15	0.95	0.94	0.0042	-0.003	10.74%	8.66%
6653	Eildon	AU.	3.39	0.92	0.93	0.075	0.17	9.27%	9.98%
6733	Copeton	AU.	1.36	0.94	0.94	-0.038	0.056	9.75%	8.22%
	Average			0.82	0.81	0.041	0.23	13.28%	24.30%

[a]Cap represents the reservoir storage capacity value, [b]Bathy represents the results from the bathymetry based method, and [c]SIM represents the results from the simulation based method.

References:

1. Yigzaw, W. et al. A New Global Storage-Area-Depth Dataset for Modeling Reservoirs in Land Surface and Earth System Models. *Water Resources Research* 54, 10,372-310,386 (2018).
