# Supplementary material for: Diminishing storage returns of reservoir construction
Source: Nat Commun. 2023 Jun 13;14:3203. doi: 10.1038/s41467-023-38843-5 (PMC10264411; doi:10.1038/s41467-023-38843-5)
Supplement: Supplementary file 2 — Peer Review File [file 41467_2023_38843_MOESM2_ESM.pdf]

nature portfolio

Peer Review File

Diminishing storage returns of reservoir construction

Open Access This file is licensed under a Creative Commons Attribution 4.0 International License, which permits use, sharing, adaptation, distribution and reproduction in any medium or format, as long as you give appropriate credit to the original author(s) and the source, provide a link to the Creative Commons license, and indicate if changes were made. In the cases where the authors are anonymous, such as is the case for the reports of anonymous peer reviewers, author attribution should be to 'Anonymous Referee' followed by a clear attribution to the source work. The images or other third party material in this file are included in the article's Creative Commons license, unless indicated otherwise in a credit line to the material. If material is not included in the article's Creative Commons license and your intended use is not permitted by statutory regulation or exceeds the permitted use, you will need to obtain permission directly from the copyright holder. To view a copy of this license, visit http://creativecommons.org/licenses/by/4.0/.

Editorial Note: This manuscript has been previously reviewed at another journal that is not operating a transparent peer review scheme. This document only contains reviewer comments and rebuttal letters for versions considered at *Nature Communications.*

REVIEWER COMMENTS

Reviewer #1 (Remarks to the Author):

I have carefully reviewed the author's revision. The authors have taken additional effort to address my technical concerns. Particularly, I was concerned about the global applicability of the authors' method in modeling reservoir bathymetric profiles. Accordingly, the authors have expanded the validation of the profile modeling method to the 347 reservoirs included in the Global Reservoir Bathymetry Dataset (GRBD). The modeled reservoir storage capacities agree overall well with the storage capacities based on reservoir bathymetries in GRBD. I was also concerned about the impacts of ignoring reservoir sedimentation on the derived Normalized Storage (NS) trend. Accordingly, the authors expanded the discussion on the theoretical impact of reservoir sedimentation, accompanied by an experiment on four US reservoirs. Their discussion shows that ignoring sedimentation may underestimate the severity of the NS decline, but the underestimation is probably small. The authors have also included some of the validation statistics to the uncertainties of their trend estimates.

I found the authors' revision has further improved the fidelity of their data products and the reliability of their scientific results. While this is encouraging, I reserve a few technical concerns and remain skeptical about what's really behind the pattern of declining reservoir NS. In addition, I found the connotation of "diminishing returns of reservoir construction" rather implicit. This is partially because the current attribution, which is based on simple correlation analysis, has not yet sufficiently disentangled the signals of climate, reservoir construction, and operations/managements. I listed my remaining concerns below for the authors to further consider.

R2C3. Impact of reservoir sedimentation. The authors rebutted that their conclusions based on reservoir NS are not affected by the ignorance of reservoir sedimentation, because "sedimentation tends to reduce the actual storage (numerator) and storage capacity (denominator) equally". I agree with this rebuttal, if we assume sediment infilling did not affect the states of reservoir surface water elevation or area in the first place. This assumption may be true when the total sediment in a reservoir does not exceed the sediment reserve or the dead pool storage. But in other cases, substantial sediment infilling might lead to an altered operation of the reservoir water stage, which consequentially affected the reservoir water area. For instance, the reservoir conservation pool allows some flexibility to elevate the water level to compensate for the loss of actual water storage due to infilling, although this depends on the sedimentation adaptation policy of the reservoir. I am not sure how common this case occurred in aging reservoirs. Can the authors double check from the literature or at least include some discussion? In summary, I am concerned that, if the average water level of an aging reservoir was elevated as a response to sedimentation, the water area time series mapped from spectral images would also be enlarged, which could lead to an overestimated phase (or a positive bias) of the NS time series in the pre-1999 reservoirs.

R2C7. Validation of the reservoir bathymetric profile models. This expanded validation, which included 347 reservoirs with detailed bathymetry in GRBD, still focuses on total reservoir storage capacity, NOT the NS trend that matters more to the authors' scientific conclusions. Since the profile model selection was gauged by storage capacity, it is not too surprising that the modeled storage capacities agree well with those in GRBD. However, I am not sure the agreement in storage capacity also warrants a good agreement between the NS trends derived from the two methods. As I suggested previously, I strongly encourage the authors to go further and conduct a more comprehensive comparison between the results (not only storage capacities but also NS trends) based on the profile modeling method and the results based on GRBD bathymetries. I see this additional validation very important for justifying and assuring a global applicability of the authors' modeling method.

R2C10. Reservoir water area at capacity. Based on the equations in Extended Data Table 2, the water area at capacity (Ao) functions as a scaling factor and is critical to the accuracies of the derived storage change and NS time series. In general, an overestimated Ao would lead to a

negative bias of the NS time series, and vice versa. For this reason, I have been critical about whether the authors estimated Ao for pre-1999 and post-1999 reservoirs consistently, and particularly, whether there is a possibility that the values of Ao for pre-1999 reservoirs were "underestimated" in comparison with those for post-1999 ones. If so, the higher phase of the NS time series in the pre-1999 reservoirs (a key finding of this paper) would be, in part, an artefact of the scaling bias.

As the authors clarified in the rebuttal, "if the 95th percentile area was less than the reported area in GRanD, the maximum documented area value was selected to represent the area at capacity", and "only 20% of the post-1999 reservoirs use the 95-percentile area to represent the area at capacity". This means that for the majority (80%) of post-1999 reservoirs, the maximum area value documented in GRanD is greater than the 95-percentile area in GRSAD. This is in contrary to pre-1999 reservoirs, where the majority (roughly 75%) ended up having the value in GRanD replaced by the 95-percentile area in GRSAD to present the area at capacity (as described in Lines 263-266). This means that the maximum area available in GRanD is smaller than the 95-percentile area in GRSAD for most of the pre-1999 reservoirs.

By looking into GRanD v1.3, I noticed that for about 50% of the post-1999 reservoirs, the maximum area value in GRanD comes from the polygon area, rather than "Area_rep" or "Area_max". As described in the technical documentation of GRanD, most new reservoir polygons added in v1.3 were based on the maximum (100-percentile) water occurrence extent of the Joint Research Center (JRC) product (Pekel et al. 2016). This perhaps explained why the authors here found the GRanD areas for most post-1999 reservoirs are already larger than the 95-percentile GRSAD areas (which were also based on the JRC occurrence product). Regardless of the reason, we here may have Ao for most pre-1999 reservoirs based on the 95-percentile area but Ao for most post-1999 reservoirs based on the water extent larger than the 95-percentile area. This inconsistency echoes my above-described concern, i.e., the decrease of NS in post-1999 reservoirs as a false signal. I recommend the authors to further examine this possibility.

Eventually, about the meaning of "diminishing returns of reservoir construction". I still struggle to fully understand what this expression conveys scientifically, so I highly recommend that the authors explicitly define the connotation of this expression in text. Even if the above technical concerns are all cleared, the relatively low NS value and the declining NS trend in post-1999 reservoirs may not necessarily suggest new reservoir constructions are diminishing. As seen in Figure 1, post- and pre-1999 reservoirs exhibit very different spatial distributions: as the authors also mentioned, post-1999 reservoirs are more spread out whereas newer reservoirs tend to emerge in developing countries, with a major cluster in the headwater regions of southwestern China and Southeast Asia. It is completely possible that this geographic skewedness, with differences in stream power, climate, and reservoir operation rules, may have contributed to part of the difference in NS behaviors (phase, trend, and seasonal amplitude) between pre- and post-1999 reservoirs. Additionally, the existing attribution analysis also indicates the NS declines in South America and Africa were probably climate-related, rather than being an impact of new reservoirs. So overall, some of the claims, such as "reservoirs constructed in the 21st century" are "more susceptible to droughts" and "the recent returns of global reservoir constructions have been declining", appear vague and lacking logical supports.

Reviewer #2 (Remarks to the Author):

The authors have carefully addressed my previous comments. I reiterate the value of this study. However, while I appreciate that the authors added caveats regarding sedimentation, the analysis and additional elements provided in response to my comments and that of the other reviewer warrant adjustment.

First, P20L362 "Despite the impacts of sedimentation on the absolute reservoir storage, the conclusions based on the normalized storage (NS) results are not affected. This is because sedimentation tends to reduce the actual storage (numerator) and storage capacity (denominator) equally, so the NS is not much affected." Although the statement that NS is unaffected is valid, this strict definition of NS (using current storage capacity rather than initial storage capacity as the denominator) is of little relevance for management. What really matters for water provision and hydroelectric production for example, is actual storage/initial storage capacity. Therefore, I suggest removing this argument.

Second, I question the choice of reservoirs for illustrating the impact of sedimentation. How were these reservoirs chosen? The annual sedimentation rates, which range between 0.025 and 0.253%/year are on the very low range compared to global averages. The following paragraph from Wisser et al. (2013) illustrates this point : "A global data set with consistently measured sediment volumes trapped in reservoirs over time is not available, and, in fact there has been a decline in the collection of sedimentation data [Syvitski, 2003; Vanmaercke et al., 2011b]. For the US, the Reservoir Sedimentation Database (RESSED) [Ackermann et al., 2009] provides statistics on reservoir volumes derived from bathymetric surveys for more than 1800 reservoirs from which sedimentation loss rates can be calculated (using successive surveys). We calculated sedimentation rates of 1024 reservoirs with at least two complete surveys (using the most recent surveys) and supplemented this data set with observed loss rates from an additional 191 reservoirs globally from a variety of sources (Figure 1) to get a global sample of observed loss rates.This data set covers a wide range of physical conditions in the catchment (e.g., slope, climate, soil type, and land use) and local reservoir conditions (size, area, bathymetry, purpose, and management practices). Observed loss rates vary between 0.0017% a^{-1} and 36% a^{-1} and are 1.35% a^{-1} on average (median = 0.55% a^{-1}). Loss rates for reservoirs with a capacity larger than the minimum capacity in the GRanD database (1 million m3) have lower loss rates (mean = 0.76% a^{-1}, median 0.35% a^{-1}; Table 1). Weighted by initial capacity, the mean loss rate is 0.66%.
Out of fifty reservoirs in Italy, Patro et al. (2022) mention "According to the latest report of the International Commission On Large Dams (ICOLD, 2009), the global average value for sedimentation rate (and so storage loss) is around 0.96%." and find mean annual sedimentation rates of 0.57% and a total mean loss of reservoir capacity of 37%.

These points do not lessen the merit of this study but must be properly acknowledged. Sedimentation is a widespread phenomenon in reservoirs that surely has, on aggregate, more long-term impact on storage capacity than the changes observed by this study.

Once these corrections are made, I consider this study to be ready for publication.

References

Patro ER, De Michele C, Granata G, Biagini C. 2022. Assessment of current reservoir sedimentation rate and storage capacity loss: An Italian overview. Journal of Environmental Management 320: 115826.

Wisser D, Frolking S, Hagen S, Bierkens MFP. 2013. Beyond peak reservoir storage? A global estimate of declining water storage capacity in large reservoirs. Water Resources Research 49: 5732–5739.

We sincerely appreciate the constructive comments and suggestions from the reviewers and the editor. We have revised the manuscript and addressed these comments point by point. We hope this revised manuscript fulfills the editor's and reviewers' high standards for *Nature Communications*. The reviewers' comments are shown in black, our responses are highlighted in blue, and the revised text in the manuscript is highlighted in orange.

Reviewer #1 (Remarks to the Author):

I have carefully reviewed the author's revision. The authors have taken additional effort to address my technical concerns. Particularly, I was concerned about the global applicability of the authors' method in modeling reservoir bathymetric profiles. Accordingly, the authors have expanded the validation of the profile modeling method to the 347 reservoirs included in the Global Reservoir Bathymetry Dataset (GRBD). The modeled reservoir storage capacities agree overall well with the storage capacities based on reservoir bathymetries in GRBD. I was also concerned about the impacts of ignoring reservoir sedimentation on the derived Normalized Storage (NS) trend. Accordingly, the authors expanded the discussion on the theoretical impact of reservoir sedimentation, accompanied by an experiment on four US reservoirs. Their discussion shows that ignoring sedimentation may underestimate the severity of the NS decline, but the underestimation is probably small. The authors have also included some of the validation statistics to the uncertainties of their trend estimates.

I found the authors' revision has further improved the fidelity of their data products and the reliability of their scientific results. While this is encouraging, I reserve a few technical concerns and remain skeptical about what's really behind the pattern of declining reservoir NS. In addition, I found the connotation of "diminishing returns of reservoir construction" rather implicit. This is partially because the current attribution, which is based on simple correlation analysis, has not yet sufficiently disentangled the signals of climate, reservoir construction, and operations/managements. I listed my remaining concerns below for the authors to further consider.

We greatly appreciate your comments. They have significantly improved the manuscript. We have addressed them in the following responses, and have revised the manuscript accordingly.

R2C3. Impact of reservoir sedimentation. The authors rebutted that their conclusions based on reservoir NS are not affected by the ignorance of reservoir sedimentation, because "sedimentation tends to reduce the actual storage (numerator) and storage capacity (denominator) equally". I agree with this rebuttal, if we assume sediment infilling did not affect the states of reservoir surface water elevation or area in the first place. This assumption may be true when the total sediment in a reservoir does not exceed the sediment reserve or the dead pool storage. But in other cases, substantial sediment infilling might lead to an altered operation of the reservoir water stage, which consequentially affected the reservoir water area. For instance, the reservoir conservation pool allows some flexibility to elevate the water level to compensate for the loss of actual water storage due to infilling, although this depends on the sedimentation adaptation policy of the reservoir. I am not sure how common this case occurred in aging reservoirs. Can the authors double check from the literature or at least include some discussion? In summary, I am concerned that, if the average water level of an aging reservoir was elevated as a response to sedimentation, the water area time series mapped from spectral images would also be enlarged, which could lead to an overestimated phase (or a positive bias) of the NS time series in the pre-1999 reservoirs.

Thanks for this comment. We have searched the literature broadly (e.g., journal papers and technical reports) but did not find explicit answers for this comment. Therefore, to find out how common this case occurs in aging reservoirs affected by sedimentation, we consulted Dagmar Llewellyn, a hydrologist and planning supervisor from Bureau of Reclamation. Such cases should be very rare, according to her response below:

"Water managers do not often change their operations in response to sediment build up in the reservoirs, and that sediment does not often change the surface area of the reservoir when it is full. There are certain circumstances in which what the reviewer is saying could occur. For example, if a flood-control reservoir has a recreation pool for which Congress authorized a certain volume of water, which is well below the capacity of the reservoir. In this case, yes, sedimentation could increase the surface area required to store the specified amount of water. However, I think this in an exception that proves the rule that, for most reservoirs, this would not happen - we water managers would not change our operation because of sediment at the bottom of the reservoir. I think this would almost never be done for water storage reservoirs, since generally, these reservoirs store whatever water is available, up to the capacity of the reservoir.

Sediment at the bottom of the reservoir does not allow for water to be stored higher, since the total storage is limited by the dam. Instead, the capacity of the reservoir just decreases for the same surface area. For flood control reservoirs, there is some small possibility (like in my first example), but generally the flood pools are based on specific pool elevations, which correlate with a specific surface area that does not change with accumulation of sediment at the bottom of the reservoir."

R2C7. Validation of the reservoir bathymetric profile models. This expanded validation, which included 347 reservoirs with detailed bathymetry in GRBD, still focuses on total reservoir storage capacity, NOT the NS trend that matters more to the authors' scientific conclusions. Since the profile model selection was gauged by storage capacity, it is not too surprising that the modeled storage capacities agree well with those in GRBD. However, I am not sure the agreement in storage capacity also warrants a good agreement between the NS trends derived from the two methods. As I suggested previously, I strongly encourage the authors to go further and conduct a more comprehensive comparison between the results (not only storage capacities but also NS trends) based on the profile modeling method and the results based on GRBD bathymetries. I see this additional validation very important for justifying and assuring a global applicability of the authors' modeling method.
Thanks for this comment. We compared the NS trend values derived from the simulation method with the results from bathymetry-based methods for 347 global reservoirs. Figure R1 shows that the NS trend values derived from these two methods show good agreement ($R^2 = 0.76$), with the majority of the points centered on the 1:1 line (with a slope value of 0.99).

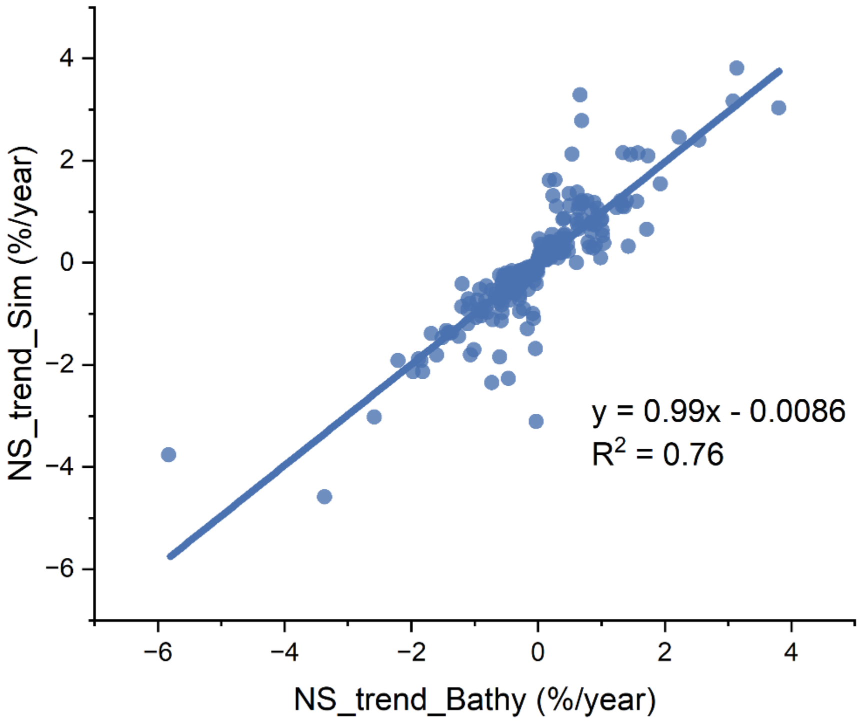

Figure R1. Comparison of NS trend values derived from bathymetry and simulation based methods.

R2C10. Reservoir water area at capacity. Based on the equations in Extended Data Table 2, the water area at capacity (Ao) functions as a scaling factor and is critical to the accuracies of the derived storage change and NS time series. In general, an overestimated Ao would lead to a negative bias of the NS time series, and vice versa. For this reason, I have been critical about whether the authors estimated Ao for pre-1999 and post-1999 reservoirs consistently, and particularly, whether there is a possibility that the values of Ao for pre-1999 reservoirs were "underestimated" in comparison with those for post-1999 ones. If so, the higher phase of the NS time series in the pre-1999 reservoirs (a key finding of this paper) would be, in part, an artefact of the scaling bias.

As the authors clarified in the rebuttal, "if the 95th percentile area was less than the reported area in GRanD, the maximum documented area value was selected to represent the area at capacity", and "only 20% of the post-1999 reservoirs use the 95-percentile area to represent the area at capacity". This means that for the majority (80%) of post-1999 reservoirs, the maximum area value documented in GRanD is greater than the 95-percentile area in GRSAD. This is in contrary to pre-1999 reservoirs, where the majority (roughly 75%) ended up having the value in GRanD

replaced by the 95-percentile area in GRSAD to present the area at capacity (as described in Lines 263-266). This means that the maximum area available in GRanD is smaller than the 95-percentile area in GRSAD for most of the pre-1999 reservoirs.

By looking into GRanD v1.3, I noticed that for about 50% of the post-1999 reservoirs, the maximum area value in GRanD comes from the polygon area, rather than "Area_rep" or "Area_max". As described in the technical documentation of GRanD, most new reservoir polygons added in v1.3 were based on the maximum (100-percentile) water occurrence extent of the Joint Research Center (JRC) product (Pekel et al. 2016). This perhaps explained why the authors here found the GRanD areas for most post-1999 reservoirs are already larger than the 95-percentile GRSAD areas (which were also based on the JRC occurrence product). Regardless of the reason, we here may have Ao for most pre-1999 reservoirs based on the 95-percentile area but Ao for most post-1999 reservoirs based on the water extent larger than the 95-percentile area. This inconsistency echoes my above-described concern, i.e., the decrease of NS in post-1999 reservoirs as a false signal. I recommend the authors to further examine this possibility.

Thanks for pointing this out. We agree with the reviewer that water area at capacity (Ao) is an important input for the simulation model. It is challenging to validate the water area values due to the lack of in situ data (Zhao et al. 2018). Therefore, we used the *in situ* storage values of the 277 reservoirs (that we used to validate our storage dataset) to conduct an uncertainty analysis, using the 95-percentile area as the water area at capacity. We used the improved simulation method to derive the storage values from 1999 to 2018, with water-area-at-capacity inputs values from the 95- and 100-percentile areas, respectively. Validation results (Figure R2a-b) indicate that the storage values from the 95- and 100-percentile area inputs agree well with the *in situ* values (R^2 values of 0.98 and 0.99, and RMSE values of 0.30 km^3 and 0.29 km^3). In addition, we compared the storage values derived from these two inputs (Figure R2c), which show good agreement (with a slope of 1.0 and a bias of 0.015 km^3). This small bias should have very limited impact on the NS values at large scales, as compared to the large capacity values. Thus, our key findings are reliable by using the 95-percentile area as the water area at capacity.

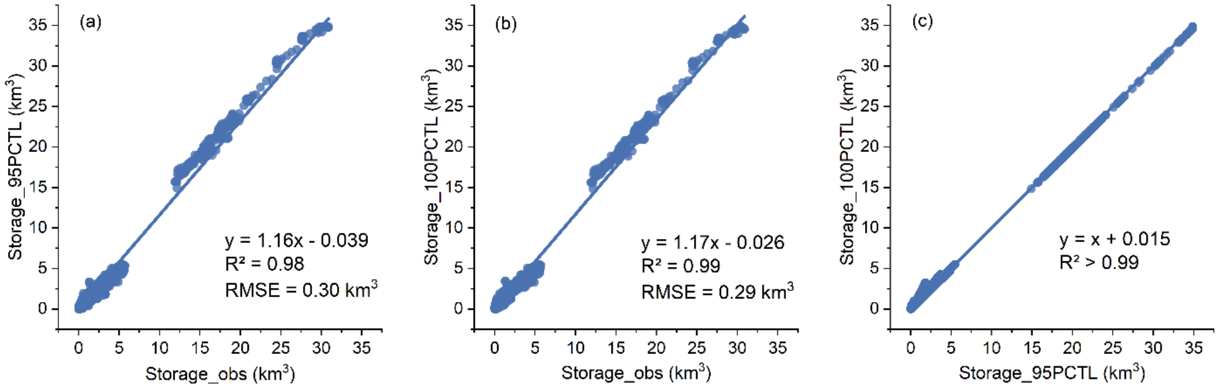

Figure R2. (a-b) Validation of simulated storage values from 1999 to 2018 with inputs of water area at capacity values from 95- and 100-percentile areas, respectively; (c) Comparison of the simulated storage values derived from these two inputs.

Reference:

Zhao, G. & Gao, H. Automatic correction of contaminated images for assessment of reservoir surface area dynamics. Geophysical Research Letters 45, 6092-6099 (2018).

Eventually, about the meaning of "diminishing returns of reservoir construction". I still struggle to fully understand what this expression conveys scientifically, so I highly recommend that the authors explicitly define the connotation of this expression in text. Even if the above technical concerns are all cleared, the relatively low NS value and the declining NS trend in post-1999 reservoirs may not necessarily suggest new reservoir constructions are diminishing. As seen in Figure 1, post- and pre-1999 reservoirs exhibit very different spatial distributions: as the authors also mentioned, post-1999 reservoirs are more spread out whereas newer reservoirs tend to emerge in developing countries, with a major cluster in the headwater regions of southwestern China and Southeast Asia. It is completely possible that this geographic skewedness, with differences in stream power, climate, and reservoir operation rules, may have contributed to part of the difference in NS behaviors (phase, trend, and seasonal amplitude) between pre- and post-1999 reservoirs. Additionally, the existing attribution analysis also indicates the NS declines in South America and Africa were probably climate-related, rather than being an impact of new reservoirs. So overall, some of the claims, such as "reservoirs constructed in the 21st century" are "more susceptible to droughts" and "the recent returns of global reservoir constructions have been declining", appear vague and lacking logical supports.

We thank the reviewer for their suggestion to clarify the meaning of the title “Diminishing Returns of Reservoir Construction”. There is an implied simplification involved with using the word "return". We are using the word "return" in the same way as the phrase "return on investment". In other words, the "return" (or benefit) of constructing new reservoirs, as defined by NS, has declined. Of course there are other benefits to building reservoirs (e.g., flood reduction, recreation), as well as costs (environmental, social), but we are framing the returns in terms of water storage. To provide more clarification, we have changed the title to “Diminishing Storage Returns of Reservoir Construction”, and modified the following sentence in the manuscript: “While the new reservoirs have led to a steadily increasing global storage capacity, their returns are diminished (i.e., their NS is lower) compared to those built in the 20th century.” (Lines 70-71).

Our finding about the decreasing NS values of new reservoirs is based on a comparison of the NS values of old and new reservoirs in the same basin, while assuming that climatology conditions within a basin were homogeneous. So it is reasonable to state that “The reservoirs constructed in the 21st century have smaller NS values and larger seasonal variations than those built earlier, suggesting they are more susceptible to droughts.”. In addition, we changed the term “returns” to “storage returns” for clarification: “With predicted decreasing runoff and increasing water demand, these observed diminishing storage returns of reservoir construction will likely persist into the future.” (Lines 23-25), “The contrasting storage and NS trends indicate that the recent storage returns of global reservoir construction have been declining since the start of the 21st century.” (Lines 100-102), “The results from this study highlight the challenges of resolving finite water resources through reservoir regulations, particularly in developing countries where the storage returns from these impoundments are diminishing.” (Lines 206-209).

Reviewer #2 (Remarks to the Author):

The authors have carefully addressed my previous comments. I reiterate the value of this study. However, while I appreciate that the authors added caveats regarding sedimentation, the analysis and additional elements provided in response to my comments and that of the other reviewer warrant adjustment.

First, P20L362 “Despite the impacts of sedimentation on the absolute reservoir storage, the conclusions based on the normalized storage (NS) results are not affected. This is because

sedimentation tends to reduce the actual storage (numerator) and storage capacity (denominator) equally, so the NS is not much affected." Although the statement that NS is unaffected is valid, this strict definition of NS (using current storage capacity rather than initial storage capacity as the denominator) is of little relevance for management. What really matters for water provision and hydroelectric production for example, is actual storage/initial storage capacity. Therefore, I suggest removing this argument.

Thanks for this comment. We have removed this statement.

Second, I question the choice of reservoirs for illustrating the impact of sedimentation. How were these reservoirs chosen? The annual sedimentation rates, which range between 0.025 and 0.253%/year are on the very low range compared to global averages. The following paragraph from Wisser et al. (2013) illustrates this point : "A global data set with consistently measured sediment volumes trapped in reservoirs over time is not available, and, in fact there has been a decline in the collection of sedimentation data [Syvitski, 2003; Vanmaercke et al., 2011b]. For the US, the Reservoir Sedimentation Database (RESSED) [Ackermann et al., 2009] provides statistics on reservoir volumes derived from bathymetric surveys for more than 1800 reservoirs from which sedimentation loss rates can be calculated (using successive surveys). We calculated sedimentation rates of 1024 reservoirs with at least two complete surveys (using the most recent surveys) and supplemented this data set with observed loss rates from an additional 191 reservoirs globally from a variety of sources (Figure 1) to get a global sample of observed loss rates.This data set covers a wide range of physical conditions in the catchment (e.g., slope, climate, soil type, and land use) and local reservoir conditions (size, area, bathymetry, purpose, and management practices). Observed loss rates vary between 0.0017% a^{-1} and 36% a^{-1} and are 1.35% a^{-1} on average (median = 0.55% a^{-1}). Loss rates for reservoirs with a capacity larger than the minimum capacity in the GRanD database (1 million m3) have lower loss rates (mean = 0.76% a^{-1}, median 0.35% a^{-1}; Table 1). Weighted by initial capacity, the mean loss rate is 0.66%.

Out of fifty reservoirs in Italy, Patro et al. (2022) mention "According to the latest report of the International Commission On Large Dams (ICOLD, 2009), the global average value for sedimentation rate (and so storage loss) is around 0.96%." and find mean annual sedimentation rates of 0.57% and a total mean loss of reservoir capacity of 37%.

These points do not lessen the merit of this study but must be properly acknowledged. Sedimentation is a widespread phenomenon in reservoirs that surely has, on aggregate, more long-term impact on storage capacity than the changes observed by this study.

Thanks for pointing this out. In the previous response, we randomly selected four reservoirs with available sediment rate values from the Duke Nicholas Institute (https://nicholasinstitute.duke.edu/reservoir-national-trends/sediment). This database provides sediment rate data for 150 U.S. reservoirs, and 100 of them are included in our dataset—which were selected to further evaluate the effects of sedimentation on the NS trend values. The sediment rate values of these reservoirs range from 0 to 18.18 %/year, with mean and median values of 1.37 and 0.70 %/year, respectively—which are close to the values in the above-references mentioned by the reviewer. Figure R3 shows the distribution of the sediment values, with over half of the reservoirs having sediment rates below 1 %/year.

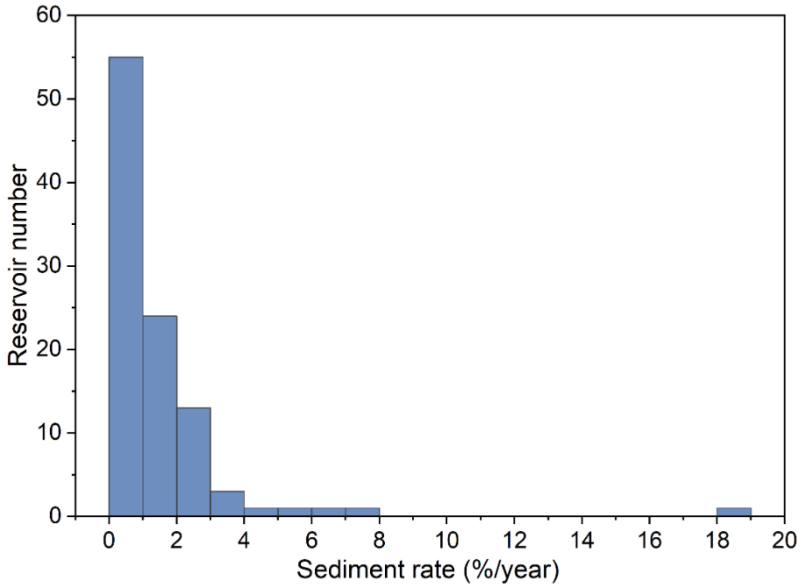

Figure R3. Distribution of the sediment rates for the 100 reservoirs used to evaluate the effects of sedimentation on the NS trends.

Figure R4 shows the comparison of the NS trend values with and without considering the sedimentation at these 100 selected reservoirs. The results suggest that sedimentation can reduce the NS trend values. Thus, the decreasing NS trends presented in this study are conservative. We did not consider the sedimentation because a reliable sedimentation database is still not available at the global scale. We have updated Extended Data Fig. 7 and added the following description

in Lines 362-368, "The sediment rate values of these reservoirs range from 0 to 18.18 %/year, with mean and median values of 1.37 %/year and 0.70 %/year, respectively (Extended Data Fig. 7a). We compared the NS trend values with and without considering the impacts of sedimentation (Extended Data Fig. 7b). This comparison shows that sedimentation can reduce the NS trend values. Note that the sediment dataset has a relatively large uncertainty due to the scarcity of field measurements and the effects of sediment removal activities (e.g., dredging and flushing)."

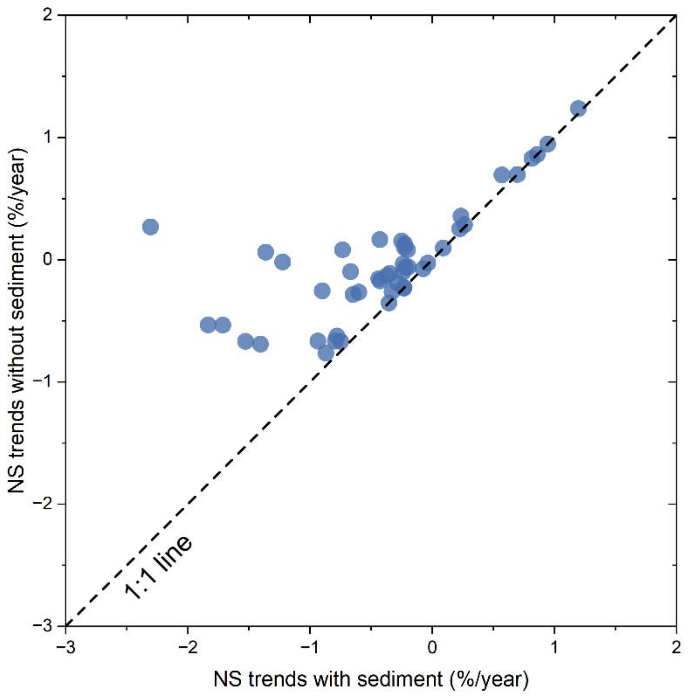

Figure R4. Comparison of NS trend values with and without considering sedimentation.

Once these corrections are made, I consider this study to be ready for publication.

Thanks again for the reviewer's time and effort in helping to improve the manuscript.

References

Patro ER, De Michele C, Granata G, Biagini C. 2022. Assessment of current reservoir sedimentation rate and storage capacity loss: An Italian overview. Journal of Environmental Management 320: 115826.

Wisser D, Frolking S, Hagen S, Bierkens MFP. 2013. Beyond peak reservoir storage? A global estimate of declining water storage capacity in large reservoirs. Water Resources Research 49: 5732–5739.

REVIEWER COMMENTS

Reviewer #1 (Remarks to the Author):

I want to thank the authors for their continued effort improving the manuscript. I have carefully reviewed their revision for this round, and found my major concerns addressed fairly well and the manuscript further improved. I have a few more suggestions based on the authors' responses to each of my previous concerns. Once these suggestions have been incorporated, I will feel comfortable to recommend its publication in Nature Communications.

About sedimentation impact on reservoir water level and area. I would like to thank the authors for looking into the literature and consulting Dagmar Llewellyn in the Bureau of Reclamation. I accept this rebuttal. Since potential readers may have similar concerns, I highly recommend that the authors include a similar discussion in the paper (in either the main text or methods) to echo this rebuttal.

For example, the authors can briefly discuss: (1) how reservoir management may respond to sedimentation (in most cases, sedimentation does not allow for water to be stored higher, with the exception of some of the flood-control reservoirs), and (2) how this response implies to the validity of the authors' methods and conclusions (as sediment accumulates, the reservoir storage capacity may just decrease under the same water area, leading to limited impacts on the NS).

About validation of the NS trends. It seems the NS trends derived from the simulation method are overall comparable with the trends from the bathymetry-based method. Although there are some disagreement and variability, the mean bias between the two methods appears low. I think this is a positive message. I highly recommend the authors add this validation (with Figure R1) to the paper (such as in "Evaluation of storage estimation" section), because given the focus here, validating NS trends is just as important as validating storage capacities. As another (optional) suggestion: If I were the authors, I would also try to absorb the validation statistics (such as the RMSE in Figure R1) to the uncertainties of the NS trend estimates for at least the 347 GRBD reservoirs.

About reservoir water area at capacity (Ao). It is good to see the authors have performed this sensitivity test and the difference between the estimated capacities using 95% Ao and those using 100% Ao appears limited based on the 277 reservoirs with in-situ storage. This is encouraging. To further secure this very important aspect, I am wondering if the authors can expand this sensitivity analysis to other reservoirs, in order to inform the potential impact of inconsistent use of Ao on the conclusions about pre- and post-1999 NS.

For example, I suggest the following scheme for the sensitivity analysis (which should be straightforward to implement): (1) for the subset of pre-1999 reservoirs using 95% water occurrence as Ao, replace their Ao by 100% water occurrence, (2) for the subset of post-1999 reservoirs using 95% water occurrence as Ao, keep their Ao as they are (basically do nothing for post-1999 reservoirs), and (3) compare how much difference this inconsistent use of Ao will cause for the calculated pre-1999 and post-1999 NS values. This design should, in theory, report the maximum impact of Ao. As a reminder, the authors may need to re-simulate the geometric models for some of the pre-1999 reservoirs because their Ao is changed. I also recommend that the authors include such a sensitivity analysis in the method section, which will secure to readers that this aspect won't affect the main conclusions of the paper.

About "diminishing returns". I want to thank the authors for clarifying the meaning of "return" and making modifications in the text. While I am still not fully convinced by some of the arguments, the changes have been much helpful and clearer. My concern is: even the same basin does not warrant a homogenous hydroclimate condition, and reservoir operation and storage pattern vary by stream power, geomorphology, and nuances in water management, which can vary reservoir by reservoir and across the same basin. So as a reader, I am not fully convinced that smaller NS values and larger seasonal variations in the post-1999 reservoirs have to suggest newer reservoirs are more susceptible to drought and brining less "return" values. I recommend that the authors give a last try to further clarity some of the terms (such as explicitly define what "return" means

as early as possible) and probably soften some of the claims lacking solid supports.

Reviewer #2 (Remarks to the Author):

The authors have expanded great efforts in addressing all of the reviewers' concerns and suggestions over all rounds of review, which I applaud. I do not have additional comments.

Best wishes,
Mathis L. Messager

We sincerely appreciate the constructive comments and suggestions from the reviewers and the editor. We have revised the manuscript and addressed these comments point by point. We hope this revised manuscript fulfills the editor's and reviewers' high standards for *Nature Communications*. The reviewers' comments are shown in black, our responses are highlighted in blue, and the revised text in the manuscript is highlighted in orange.

Reviewer #1 (Remarks to the Author):

I want to thank the authors for their continued effort improving the manuscript. I have carefully reviewed their revision for this round, and found my major concerns addressed fairly well and the manuscript further improved. I have a few more suggestions based on the authors' responses to each of my previous concerns. Once these suggestions have been incorporated, I will feel comfortable to recommend its publication in Nature Communications.
We thank the reviewer for their time and effort in helping us to improve the manuscript.

About sedimentation impact on reservoir water level and area. I would like to thank the authors for looking into the literature and consulting Dagmar Llewellyn in the Bureau of Reclamation. I accept this rebuttal. Since potential readers may have similar concerns, I highly recommend that the authors include a similar discussion in the paper (in either the main text or methods) to echo this rebuttal.
For example, the authors can briefly discuss: (1) how reservoir management may respond to sedimentation (in most cases, sedimentation does not allow for water to be stored higher, with the exception of some of the flood-control reservoirs), and (2) how this response implies to the validity of the authors' methods and conclusions (as sediment accumulates, the reservoir storage capacity may just decrease under the same water area, leading to limited impacts on the NS).
Thanks for this comment. We have added discussion in the Methods section about the impact of sedimentation on reservoir water level and area changes (Lines 367-376): "Although sedimentation can reduce storage capacity, reservoir management does not allow for water to be stored higher due to concerns about dam safety. One rare exception is when a flood control reservoir has a recreation pool for which a certain volume of water is required to be maintained, and the surface level (of this required volume) is well below the elevation at capacity. In this

case, sedimentation can increase the surface area required to store the specified amount of water. Flushing or dredging is usually applied for mitigating the sedimentation effects on storage. In this study, our method relies on using surface area to infer the storage and does not consider the sedimentation effects. If the sedimentation amount is notable in a reservoir, then both the storage and storage capacity can be overestimated. However, based on the evaluation below, sedimentation has a limited effect on the NS values and the NS trends."

The new text focuses on point (1). For point (2), we have included a detailed explaination that addresses the observed sedimentation data (Lines 377-393).

About validation of the NS trends. It seems the NS trends derived from the simulation method are overall comparable with the trends from the bathymetry-based method. Although there are some disagreement and variability, the mean bias between the two methods appears low. I think this is a positive message. I highly recommend the authors add this validation (with Figure R1) to the paper (such as in "Evaluation of storage estimation" section), because given the focus here, validating NS trends is just as important as validating storage capacities. As another (optional) suggestion: If I were the authors, I would also try to absorb the validation statistics (such as the RMSE in Figure R1) to the uncertainties of the NS trend estimates for at least the 347 GRBD reservoirs.

Thanks for this comment. We have added language about this validation and a new figure (Supplementary Fig. 7) to the "Evaluation of storage estimation" section (Lines 345-349), "In addition, we compared the NS trend values derived from the simulation method with the results from bathymetry-based methods for the 347 global reservoirs. Supplementary Fig. 7 shows that the NS trend values derived from these two methods show good agreement (R^2 = 0.76), with the majority of the data aligning with the 1:1 line (with a slope value of 0.99)."

As the NS trends are primarily evaluated at large scales in this study, we have used the validation statistics in Supplementary Fig. 5 to assess the uncertainties of the NS trends.

About reservoir water area at capacity (Ao). It is good to see the authors have performed this sensitivity test and the difference between the estimated capacities using 95% Ao and those using 100% Ao appears limited based on the 277 reservoirs with in-situ storage. This is encouraging. To further secure this very important aspect, I am wondering if the authors can expand this

sensitivity analysis to other reservoirs, in order to inform the potential impact of inconsistent use of Ao on the conclusions about pre- and post-1999 NS.

For example, I suggest the following scheme for the sensitivity analysis (which should be straightforward to implement): (1) for the subset of pre-1999 reservoirs using 95% water occurrence as Ao, replace their Ao by 100% water occurrence, (2) for the subset of post-1999 reservoirs using 95% water occurrence as Ao, keep their Ao as they are (basically do nothing for post-1999 reservoirs), and (3) compare how much difference this inconsistent use of Ao will cause for the calculated pre-1999 and post-1999 NS values. This design should, in theory, report the maximum impact of Ao. As a reminder, the authors may need to re-simulate the geometric models for some of the pre-1999 reservoirs because their Ao is changed. I also recommend that the authors include such a sensitivity analysis in the method section, which will secure to readers that this aspect won't affect the main conclusions of the paper.

We extended the uncertainty analysis to the global reservoirs. We generated another global storage dataset by using the100-percentile areas to replace the 95-percentile areas , and then compared the pre-1999 and post-1999 NS values between these two datasets. This is similar to the scheme the reviewer suggested because most of the new reservoir polygons added in v1.3 were based on the maximum (100-percentile) water occurrence extent that is equal to our 100-percentile area. Results show that the Ao values have limited impact on the pre-1999 and post-1999 NS values. We have added sensitivity analysis to the "Sources of uncertainty" section (Lines 417-435), "The water area at capacity is an important input for the simulation model. We adopted the 95-percentile areas to represent the water area at capacity for the majority of the reservoirs. The 100-percentile areas were not selected because these values may correspond to flooding circumstances, which can lead to overestimated water area values at capacity. However, most of the new reservoir polygons added in GRanD v1.3 were based on the maximum (100-percentile) water occurrence extent from the GSW dataset. Therefore, we conducted an uncertainty analysis using the 95- and 100-percentile areas as inputs. Specifically, we used the improved simulation method to derive the storage values from 1999 to 2018, with water-area-at-capacity input values from the 95- and 100-percentile areas, respectively. The *in situ* storage values of the 277 reservoirs (that we used to validate our storage dataset) were used to validate the simulated storage. Validation results (Supplementary Fig. 10a-b) indicate that the storage values from the 95- and 100-percentile area inputs agree well with the *in situ* measured values (with R^2 values of 0.98 and 0.99, and RMSE values of 0.30 km^3 and 0.29

km^3). We then compared the storage values derived from these two inputs (Supplementary Fig. 10c), which show good agreement (with a slope of 1.0 and a bias of 0.015 km^3). Additionally, we evaluated the impacts of the water-area-at-capacity inputs on the NS values. We used the 100-percentile areas to replace the 95-percentile areas to generate a different version of the global storage dataset, and then compared the pre-1999 and post-1999 NS values between these two datasets. Results show that the water-area-at-capacity inputs only have a limited impact on the NS values (Supplementary Fig. 10d-e)."

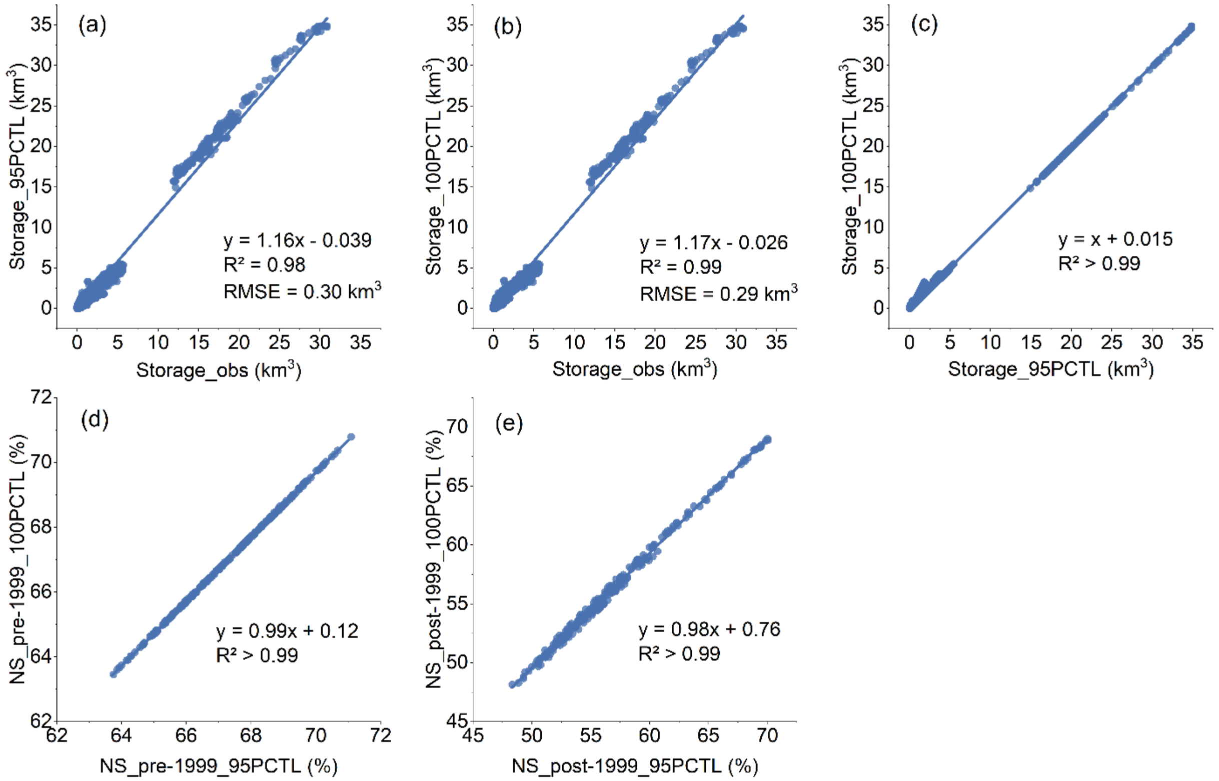

Supplementary Fig. 10 (a-b) Validation of simulated storage values from 1999 to 2018 with water area at capacity values from 95- and 100-percentile areas (respectively) used as inputs; (c) Comparison of the simulated storage values derived from these two inputs; (d-e) Comparison of NS values for pre- and post-1999 reservoirs between global storage datasets using the 95- and 100-percentile areas as water-area-at-capacity inputs.

About "diminishing returns". I want to thank the authors for clarifying the meaning of "return" and making modifications in the text. While I am still not fully convinced by some of the arguments, the changes have been much helpful and clearer. My concern is: even the same basin does not warrant a homogenous hydroclimate condition, and reservoir operation and storage

pattern vary by stream power, geomorphology, and nuances in water management, which can vary reservoir by reservoir and across the same basin. So as a reader, I am not fully convinced that smaller NS values and larger seasonal variations in the post-1999 reservoirs have to suggest newer reservoirs are more susceptible to drought and brining less “return” values. I recommend that the authors give a last try to further clarity some of the terms (such as explicitly define what “return” means as early as possible) and probably soften some of the claims lacking solid supports.

We agree with the reviewer that the hydroclimate conditions, reservoir operations, and storage patterns may vary reservoir by reservoir across the same basin. Considering these factors, we only selected groupings of reservoirs located in the same basin with the same primary function, with each basin containing at least five new reservoirs and five old reservoirs (to be spatially representative). To be clear, we did not compare the NS values of individual reservoirs. Instead, we treated the new and old reservoirs as two groups, and compared the NS values of the two groups during the same time period (1999-2018). In this way, the climate and human effects could be reduced as much as possible. We have added an explicit clarifications of “return” in Lines 70-72 and Line 80-82, “While the newly constructed reservoirs have contributed to a steadily increasing global storage capacity, their returns (in terms of NS) are found to be smaller than those built in the 20th century (over the period of 1999-2018).” “It is worth noting that there are many other social-economic benefits to building reservoirs (e.g., hydropower generation, flood reduction, water supply, and recreation), but here we are framing the returns only in terms of normalized water storage.”

Reviewer #2 (Remarks to the Author):

The authors have expanded great efforts in addressing all of the reviewers' concerns and suggestions over all rounds of review, which I applaud. I do not have additional comments.

Best wishes,
Mathis L. Messager

We greatly appreciate your comments. They have significantly improved the manuscript.

REVIEWERS' COMMENTS

Reviewer #1 (Remarks to the Author):

I want to thank the authors for taking additional time investigating the comments I suggested last time. I have reviewed the authors' responses and revision, and found them addressed well and the uncertainties acceptable. Therefore, I am overall content with and convinced by the scientific rigor and conclusions. I have no more comments. I want to congratulate the authors for producing such a nice paper!

Reviewer #1 (Remarks to the Author):

I want to thank the authors for taking additional time investigating the comments I suggested last time. I have reviewed the authors' responses and revision, and found them addressed well and the uncertainties acceptable. Therefore, I am overall content with and convinced by the scientific rigor and conclusions. I have no more comments. I want to congratulate the authors for producing such a nice paper!

We thank the reviewer for your time and effort in helping us to improve the manuscript.
